# Facilitating the measurement and treatment of Behavioral and Psychological Symptoms of Dementia (BPSD) and understanding caregiver burden using wearable devices in Rural Taiwan—Protocol for a dyadic feasibility pilot study

**DOI:** 10.1371/journal.pone.0342136

**Published:** 2026-05-18

**Authors:** Ta-Wei Guu, Wan-Jing Li, Shih-Hsiung Lee, Chih-Shan Hsu, Chia-Ni Chou, Leon Lack, Wei-Fen Ma

**Affiliations:** 1 Division of Psychiatry, Departments of Internal Medicine, China Medical University Beigang Hospital, Yunlin, Taiwan; 2 Centre for Healthy Brain Ageing, Institute of Psychiatry, Psychology and Neuroscience, King’s College London, London, United Kingdom; 3 Department of Nursing, Asia University, Taichung, Taiwan; 4 Division of Neurology, Departments of Internal Medicine, China Medical University Beigang Hospital, Yunlin, Taiwan; 5 Community Longterm Care Center, China Medical University Beigang Hospital, Yunlin, Taiwan; 6 School of Psychology, Flinders University of South Australia, Adelaide, South Australia, Australia; 7 School of Nursing, China Medical University, Taichung, Taiwan; 8 Departments of Nursing, China Medical University Hospital, Taichung, Taiwan; Shimonoseki City University, JAPAN

## Abstract

**Introduction:**

Alzheimer’s disease (AD) prevalence rises with societal ageing. In clinical care, behavioral and psychological symptoms of dementia (BPSD)—including depression, agitation/aggression, apathy, and sleep disturbance—worsen patients’ quality of life and substantially increase caregiver burden, more significantly than the cognitive symptoms. Standard BPSD assessments rely on caregiver-rated questionnaires that are cross-sectional and may be biased when caregivers are themselves older adults. Device-based measures (e.g., research-grade wrist actigraphy) can provide objective longitudinal data and novel features. In parallel, therapeutic wearables may improve sleep and mood in adults, and might improve BPSD if accepted by people living with dementia.

**Methods:**

This dyadic pilot study will recruit 20 participants (n = 10 AD patients; n = 10 caregivers) from outpatient services and affiliated day-care/dementia hubs in rural Taiwan. Participants will wear Geneactiv continuously for 8 weeks and Re-Timer ≥30 min/day for 4 weeks. Device-based data will be processed with GGIR, a well validated R-package designed for processing accelerometer data. Questionnaire assessments include Pittsburgh Sleep Quality Index (PSQI), Neuropsychiatry Inventory Questionnaire (NPI-Q), Caregiver Burden Inventory (CBI), and a semi-structured interview based on the Taiwanese version of Quebec User Evaluation of Satisfaction with Assistive Technology (T-QUEST) at prespecified timepoints.

**Discussion:**

Wearable devices may facilitate the measurement and treatment of specific BPSD, as well as reduce caregiver burden. If proven feasible even in rural Taiwan where both digital and health literacy and resources are limited, this model will inform how device-based dementia care model can be considered and applied in the context of global ageing.

## 1. Introduction

Globally, the number of people living with dementia has been projected to increase to 153 million by 2050 [1], and will result in huge societal cost. As Taiwan entering a “super-aged society” (≥20 percent of the population aged ≥65 years old), the rapidly rising AD cases and reduced younger population substantially increase care demands, especially in rural areas [2]. AD patients frequently present with behavioral and psychological symptoms (BPSD) [3,4]. Among all the BPSD symptoms, depression, agitation/aggression, apathy, and nocturnal sleep problems often consume the most care resources and markedly elevate caregiver stress and sleep disturbances [5,6].

Current assessment of BPSD depends on clinical interviews and caregiver-completed questionnaires such as the Neuropsychiatry Inventory (NPI) and Neuropsychiatry Inventory Questionnaire (NPI-Q) [7,8], which may be imprecise given caregiver age-related declines in memory and verbal function, especially in a super-aged society context. Device-based measurement (using research-grade actigraphy such as Geneactiv) can capture objective, longitudinal activity, sleep, and light-exposure patterns [9–11], and its derived metrics (e.g., sleep regularity index, SRI) have clinical associations with both physical and mental health outcomes [12–14].

On the treatment side, pharmacotherapy remains a common modality for BPSD despite limited approvals and known risks such as falls and mortality [15]. Non-pharmacological approaches are therefore important and are recommended by guidelines as the first line treatment [16,17]. Light plays an important role in human life, and can be delivered using wearable devices with therapeutic potential [18]. Bright-light interventions, for example, when delivered via ambient systems show mixed efficacy in people living with dementia, potentially due to distance from the eye and reduced light reception capacity in this population [19]. Re-Timer is a wearable light-emitting circadian regulating goggle (500-nm blue-green light) with preliminary evidence for effects on mood, circadian phase, and sleep in adults and older adults [20–22]. If acceptable to people living with dementia and their caregivers, it may alleviate BPSD and reduce caregiver burden.

To the best of our knowledge, no previous studies have investigated the feasibility of using wearable devices for both measuring and treating BPSD, and using the same devices to monitor and alleviate the caregiver burden in parallel. While Geneactiv is well-validated, its feasibility within rural Taiwanese dementia dyadic care remains unestablished. Unlike prior short-term studies (typically ≤4 weeks), we extend monitoring to eight weeks to assess longitudinal feasibility and capture real-world dyadic fluctuations between BPSD and caregiver stress.

To evaluate these possibilities, this study integrates two distinct wearable devices in a complementary framework: Geneactiv serves as the continuous monitoring component, while Re-Timer provides the active intervention component. Specifically, Geneactiv is utilized as an objective tool to longitudinally record participants’ light exposure, sleep, and activity to establish a baseline and track behavioral fluctuations; Re-Timer serves as a standardized green light intervention designed to modulate those same circadian parameters. This dual-device approach is necessary to capture the complex interplay between objective behavioral data and a wearable intervention within the caregiver-patient dyad, allowing us to see if the monitoring and treatment tools are mutually compatible in a home-care setting. Crucially, while the Re-Timer is a device designed to reprogram the body’s circadian rhythms, this study focuses mainly on the feasibility and acceptability of its implementation within this dyadic framework rather than providing a formal evaluation of its clinical treatment effects. Our primary aim is to apply a mixed-methods approach to assess the feasibility and acceptability of Geneactiv (a well-validated research-grade actigraphy [9,23,24] and Re-Timer for this dyadic intervention among Alzheimer’s disease (AD) patients with significant BPSD and their caregivers in rural Taiwan. Additionally, we seek to preliminarily explore the correlation between structured light intervention and both subjective and objective changes in circadian rhythms in both the people living with BPSD and their caregivers. Here we describe the design, participants, intervention, and outcome measurements of the study.

## 2. Materials and methods

### 2.1. Objectives

The primary objective of this pilot study is to determine the feasibility and acceptability of integrating Geneactiv and Re-Timer devices into the daily care routines of AD patients with significant BPSD and their caregivers in rural Taiwan. We will specifically evaluate device-wearing adherence, user satisfaction, and the practical impact of the devices on daily life. Establishing these parameters is a critical prerequisite for ensuring that high-tech wearable solutions are both functional and tolerable for this specific vulnerable population.

Subordinately, our secondary, exploratory objective is to collect preliminary data on the potential influence of these wearables on BPSD, circadian rhythmicity, and caregiver burden. These analyses are hypothesis-generating and intended to examine the relationships between light exposure and circadian metrics, as well as to compare device-based versus questionnaire-based measurements. The findings will be used to characterize potential digital biomarkers and inform the power calculations and design of future definitive clinical trials, rather than to establish treatment efficacy.

### 2.2. Study design and setting

**Fig 1:**
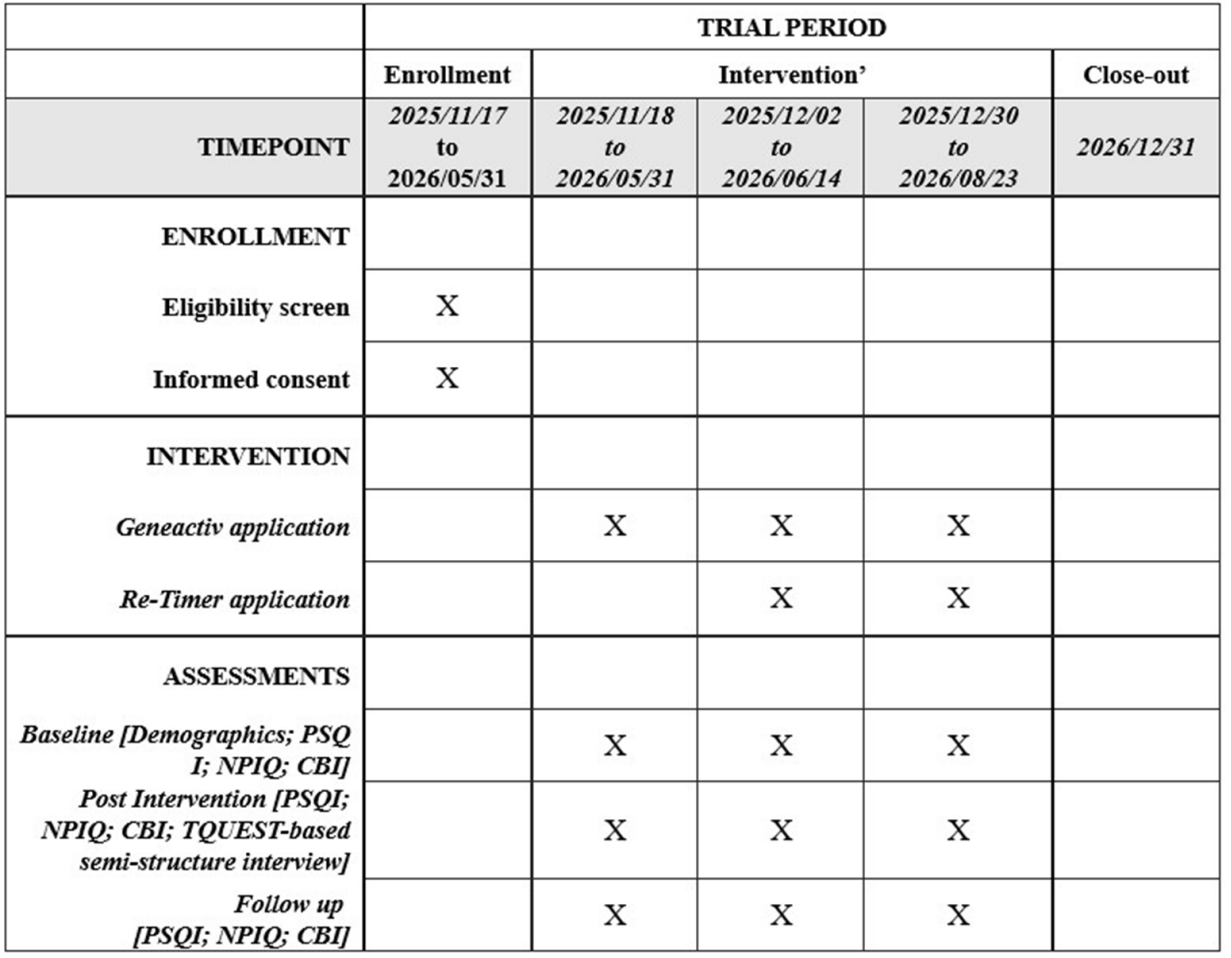
Timeline of the study. Abbreviations: PSQI = Pittsburgh Sleep Quality Index; NPIQ = Neuropsychiatric Inventory Questionnaire; CBI = Caregiver Burden Inventory; TQUEST = Taiwanese Version of the Quebec User Evaluation of Satisfaction with ‌Assistive Technology.

This is a single-arm, dyadic pilot feasibility study lasting 12 weeks, and has started recruitment on 17^th^ November 2025 in CMU Beigang Hospital outpatient psychiatry and neurology clinics, and affiliated dementia day-care center and hubs. Dyads comprise an AD/ mild cognitive impairment (MCI) due-to-AD patient with significant BPSD, and their adult primary caregiver. The recruitment and data collection will be completed in May and August 2026, respectively, while the results are expected to be available in December 2026 (see Fig 1 for the overall timeline).

#### 2.2.1. Eligibility criteria.

**Inclusion criteria for patients (probable AD or MCI due to AD):**

Participants eligible for the patient group must have a clinical diagnosis of either probable AD or MCI due to AD (CDR 0.5) per the National Institute on Aging and Alzheimer’s Association (NIA-AA) diagnostic guidelines [25]. To be included, patients must exhibit at least one significant symptom of BPSD (such as depression, agitation/aggression, apathy, and nocturnal sleep disturbance). A symptom is considered “significant” if NPI item score ≥ 4 [8] or NPI-Q item severity ≥ 2 [26]. For those whose primary BPSD symptom is sleep-related, they can be eligible either if their Pittsburgh Sleep Quality Index (PSQI) global score is ≥ 5 [27], or the sleep item of NPI/NPIQ score reaches the aforementioned thresholds.

Furthermore, patients must be residing in the same community residence or maintaining consistent day-care engagement for at least two weeks prior to enrollment. To minimize confounding effects on BPSD and sleep metrics, a parallel two-week stability requirement applies to all pharmacological and non-pharmacological treatments. Specifically, participants must be on a stable dose of psychotropic or cognitive-enhancing medications, or the same frequency of non-pharmacotherapy for at least 14 days prior to baseline assessment. The selection of a two-week window is a strategic balance consistent with published BPSD-related studies [28,29]: it provides a sufficient duration to ensure that acute adverse effects or rapid clinical responses to recent medication adjustments have stabilized, while remaining practical for a population characterized by frequent, non-linear behavioral fluctuations.

**Inclusion criteria for caregivers:**

The study also includes the adult primary caregivers of the enrolled patients. These caregivers are required to have maintained stable treatments for caregiver stress (if any, such as unchanged dose of hypnotics, or same psychotherapy/counseling frequency) for at least two weeks prior to the start of the study. All participating caregivers must be able to provide written informed consent independently.

**Exclusion criteria (patient/caregiver):**

The exclusion criteria for this study are as follows: whether patients or caregivers, will be excluded from the study if they present with conditions that increase the risk associated with bright-light exposure. Specifically, individuals with pre-existing retinal disease, those currently taking photosensitizing medications, or those who have undergone ocular surgery within the past four weeks and have not yet fully recovered are ineligible. Furthermore, any other conditions that contraindicate exposure to blue-green light, such as epilepsy, serve as grounds for exclusion. Finally, the study excludes individuals who are clinically unstable, including those experiencing acute delirium or active respiratory infections, such as COVID-19, at the time of enrollment.

### 2.3. Assessments

#### 2.3.1. Basic information collection.

For patients, the collected data include date of birth, sex, marital status, education level, timing of dementia or MCI diagnosis, and time of onset of BPSD. Current cognitive status will be documented based on the most recent assessment within six months using Clinical Dementia Rating (CDR), Cognitive Abilities Screening Instrument (CASI), or Mini-Mental State Examination (MMSE); notably, reassessment will be conducted if recent cognitive changes are suspected. As well as additional health-related data involve current dementia-related medications, smoking status, alcohol use, medical and psychiatric history, and current treatments.

Similarly, for caregivers, information on date of birth, sex, marital status, education level, smoking status, alcohol use, medical and psychiatric history, and current treatments will be gathered. If the caregiver is an older adult with possible cognitive decline, their cognitive status will also be collected (CDR, CASI, or MMSE) within six months; reassessment performed if necessary if recent changes are suspected.

#### 2.3.2. Measurements for the primary objectives.

- **Taiwanese Version of the Quebec User Evaluation of Satisfaction with Assistive Technology (T-QUEST) [30**]: The T-QUEST will assesses satisfaction with wearables and related services. There are 13 items scored from 1 (very dissatisfied) to 5 (very satisfied), and will be administered to both sides of the dyads at Day 42 (for Re-Timer) and Day 56 (for Geneactiv). If the patient cannot complete the questionnaire, only the caregiver will respond.
- **Participant Manual:** Each participant will be provided with a personalized manual. The cover will include their name, the study started date, and a QR code for immediate contact with the research team. The manual contains a plain-language introduction to the study’s objectives and tables for participants to record the daily timing and duration of Re-Timer usage and qualitative feedback regarding wearing experience. These subjective records are essential for validating the feasibility of light interventions among dementia patients and their caregivers in rural Taiwan.
- **Actigraphy Data:** Utilizing the GGIR (an R-based automated package), compliance will be assessed through raw data extracted from the Geneactiv devices. This analysis allows us to calculate precise Geneactiv wearing duration and percentage (Time worn/Total study duration) across the 8-week study period. By examining the distribution of wear time, we can evaluate the long-term acceptability of research-grade wearables in dementia patients and their caregivers in rural Taiwan. These metrics will serve as a primary indicator of whether the protocol is practical for real-world application.
  - **Semi-Structured Interviews:** The interviews will extend from T-QUEST topics to explore participants’ and caregivers’ experiences with both devices, including acceptability, ease of use, comfort, and challenges. Open questions will first be used, followed by iterative, in-depth probing that focuses on convenience, regularity, motivation, and satisfaction. Through this back‑and‑forth process, participants will be encouraged to provide everyday-life examples so that they can fully articulate their experiences and feelings when using the devices. Each interview will last approximately 30–60 minutes in a setting comfortable for participants and caregivers to ensure natural and reliable data collection. Audio recordings will be transcribed and analyzed using thematic analysis to explore perceptions of wearable devices for assessing and managing neuropsychiatric symptoms after trial participation. See supporting information for the interview guide.

#### 2.3.3. Measurements for the secondary objectives.

- **Pittsburgh Sleep Quality Index (PSQI) [27**]: A questionnaire assesses seven domains related to sleep. Each item scored 0 (no symptom) to 3. PSQI will be administered five times (Day 1, Day 15, Day 42, Day 56, Day 84) to both patients and caregivers. If the patient can complete the questionnaire independently, they will be advised to do so; otherwise, the caregiver will respond on their behalf.
- **Neuropsychiatric Inventory Questionnaire (NPI-Q) [26**]: Evaluates 12 BPSD symptoms. For each symptom: presence, severity (impact on patient, scored 1–3), and caregiver distress (impact on caregiver, scored 0–5) will be evaluated. It will be administered five times (Day 1, Day 15, Day 42, Day 56, Day 84) by the same primary caregiver throughout the study.
- **Caregiver Burden Inventory (CBI) [31**]: Assesses caregiver burden with 24 items across five domains (each scored 0–4); higher scores indicate greater burden. It will be administered three times (Day 1, Day 42, Day 84) by the same primary caregiver.

### 2.4. Device and interventions procedures

**Fig 2:**
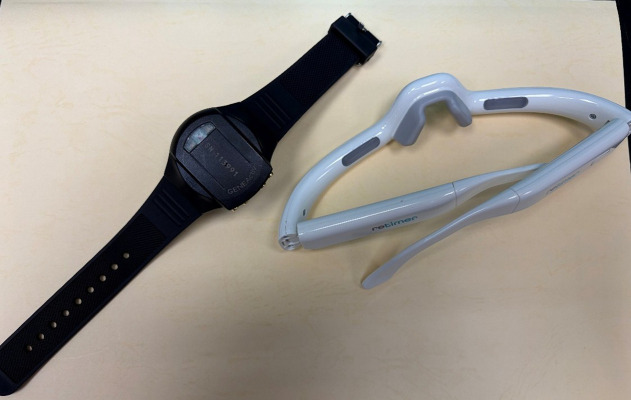
The two study devices. Left: Geneactiv research-grade actigraphy; right: Re-Timer circadian regulator. (Image taken by the authors.).

**Geneactiv wrist-worn actigraphy**: Geneactiv is a watch-like device capturing physical activities with a three-axis accelerometer (Fig 2, left). A light sensor on its surface is capable of detecting ambient illuminance. It’s feasibility and acceptability have been previously validated in dementia populations by ours and other research group independently. Based on our previous results, the team will fit devices, offer manufacturer-approved alternative straps to optimize comfort/appearance, and instruct participants to avoid covering the light sensor.

**Re-Timer circadian regulator**: Re-Timer is an eyewear emitting 500-nm blue-green visible light with no ultraviolet hazard (Fig 2, right). The device power/irradiance: 506 lux, 230 μW/cm². Theoretically, the light emitted could reduce melatonin secretion and enhance alertness. Prior studies support applicability and safety in adults, those with certain psychiatric conditions, and older adults.

**Fig 3:**
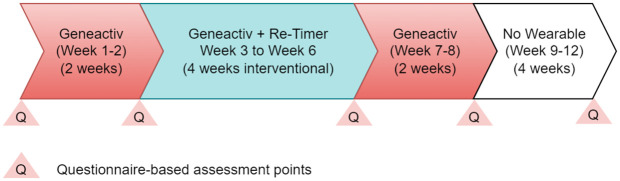
Study flow diagram. The study lasts for 12 weeks. Participants will be advised to wear the Geneactiv wrist actigraphy for up to 8 weeks, and the Re‑Timer circadian regulator for up to 4 weeks. They will complete five rounds of assessments on Day 1 (baseline before any device-wearing), Day 15 (baseline before Re-Timer), Day 42 (immediate post-Re-Timer), Day 56 (post-Re-Timer and Geneactiv), and Day 84 (trial completion).

**Device-wearing and assessment schedule** Participants will be retained in the study for 12 weeks (as in Fig 3), during which they will be advised to continue their usual daily routine/care, and will be advised to wear the device for up to 8 weeks, based on the following schedule. On Day 1, baseline demographics and initial assessments (PSQI, NPI-Q, and CBI) will be collected, and the Geneactiv monitoring will start. This is followed by Day 15, where PSQI and NPI-Q are repeated and the Re-Timer intervention begins (daily 30–60 min). On Day 42, assessments including PSQI, NPI-Q, CBI, and a T-QUEST-based semi-structured interview for Re-Timer will be conducted as the blue-green light intervention concludes. Subsequently, on Day 56, PSQI, NPI-Q, and a T-QUEST-based semi-structure interview for Geneactiv will be completed, marking the end of the Geneactiv monitoring period. Finally, a follow-up assessment of PSQI, NPI-Q, and CBI will be performed on Day 84.

More specifically on the device-wearing:

- **Geneactiv**: Days 1–56 (8 weeks) continuous wear. The device is waterproof, but participants may remove the device anytime during the trial if discomfort occurs.
- **Re-Timer**: Days 15–42 (4 weeks) ≥30 min each time, and up to 60 min/day. The participants will be recommended to wear it twice a day, one in the morning, one in early afternoon, but can remove the device and stop to use at anytime if discomfort occurs. Day-care/hub participants may choose on-site staff-assisted wear and/or home wear. Correct positioning will be taught by the study team members. A participant manual will be provided to record the device-wearing time windows each day and any feedback.

### 2.5 Study registration and ethical considerations

The study has been pre-registered in ClinicalTrials.gov with identifying number: NCT07249918 (URL: https://clinicaltrials.gov/study/NCT07249918). This protocol adheres to the Declaration of Helsinki, and has been approved by the China Medical University and Hospital Research Ethics Committee (CMUH114-REC3–072).

## 3. Outcomes assessments and data analyses

### 3.1. Primary endpoints

- **Wear compliance**: Objective Geneactiv wearing time (GGIR-derived); Re-Timer daily wear timing and duration.
- **Satisfaction (T-QUEST)** at day 42 (Re-Timer) and day 56 (Geneactiv). Between-group differences (patients vs caregivers) will be calculated by independent-samples t-tests.
- **Qualitative acceptability**: Semi-structured interviews probing convenience, comfort, barriers, and perceived value extended from the T-QUEST will be used. Transcription and thematic analysis with triangulation and independent coder checks will be done.

### 3.2. Secondary endpoints (exploratory)

- BPSD (NPI-Q) and sleep (PSQI) for patients and caregivers across five timepoints (days 1, 15, 42, 56, 84). Caregiver Burden Inventory (CBI) at days 1, 42, and 84.
- Device-based features: activity metrics; sleep metrics (sleep onset, awakenings, wake time, total sleep time, SRI); light exposure metrics; paired with NPI-Q/PSQI in time-series analyses to explore relationships among light, circadian rhythm, and BPSD.

### 3.3. Sample size and data analysis

As a pilot feasibility study, the sample size is not powered for formal hypothesis testing or clinical efficacy. A convenience sample of 10 patient–caregiver dyads (*N* = 20 participants total) is planned. This sample size is primarily determined by the primary objective of assessing recruitment and retention feasibility. Regarding the secondary exploratory objectives, this sample size is intended to provide sufficient longitudinal data points (via 8-week continuous monitoring) to estimate variance and compute preliminary effect sizes, which will serve as a basis for formal power calculations in future definitive trials.

- **Primary (Feasibility/Acceptability)**: Descriptive summaries of wear‑time compliance and T‑QUEST totals/subscales; between‑group comparisons (patients versus caregivers) via independent‑samples t‑tests as appropriate.
- **Secondary (Exploratory)**: GGIR extraction of activity, sleep (including sleep regularity index, SRI), and light; mixed‑effects models for longitudinal repeated measures with covariates (age, disease severity, comorbidities). We will evaluate potential dose–response trends between light intensity/duration and BPSD/sleep outcomes. In alignment with the exploratory nature of these endpoints, no formal p-value thresholds for efficacy will be emphasized; instead, we will compute preliminary effect sizes (Cohen’s *d*) and 95% confidence intervals to characterize the data distribution and inform future sample size planning.
- **Qualitative data analysis**: They will begin concurrently with data collection. Throughout the analytic process, peer debriefing with experienced qualitative researchers will be conducted to critically review emerging codes, categories, and themes, thereby enhancing the credibility and trustworthiness of the findings. The researchers will repeatedly review notes and audio recordings and follow these steps [32,33]:
  1. Carefully listen to the recorded interviews and transcribe them verbatim.
  2. Read the transcripts repeatedly for a comprehensive understanding, identify meaningful segments, and perform open coding to create a codebook.
  3. Apply the concept of the hermeneutic circle [32,34] to interpret experiences related to device use, focusing on aspects such as convenience, regularity, motivation, and satisfaction.
  4. Group similar meanings together.
  5. Categorize meaningful sentences and commonalities, establish categories, and name them to derive sub-themes.
  6. Re-examine the relationship between each interview’s context and the assigned categories for consistency.
  7. Reassess the core meaning of the main themes and compile an overall care framework [34].
  8. Integrate all content to produce a complete, holistic description

### 3.4. Data collection and management

All data will be pseudonymized. Unique IDs will be kept in a password-protected, encrypted network accessible only to the PI and study staff. Device fitting and troubleshooting will be documented, and adverse events will be immediately reported to the study team and logged in the participant manual. In our feasibility study, it is more crucial to ‘understand the reasons’ contributing to missing data rather than ‘deal with’ them. We will therefore describe the missing data and explore the mechanism in the semi-structured interview.

### 3.5. Safety monitoring

Participants may pause or stop device wear with any discomfort. Adverse events recorded with symptom descriptions and actions taken; referral pathways activated as needed. Serious adverse events (SAE) (e.g., hospitalization, ED visit, death) reported promptly and monitored; if three consecutive SAEs occur, the study will be temporarily halted.

### 3.6. Public and Patient Involvement (PPIE)

This study incorporates a robust Patient and Public Involvement and Engagement (PPIE) framework to ensure the current research and future studies are transparent, inclusive, and grounded in lived experience. An Advisory Group will be established, comprising study participants (patients and caregivers), trial-related clinicians, and experienced researchers. This multidisciplinary group will provide iterative consultation for study design: reviewing participant-facing materials for cultural and practical appropriateness during the design phase, addressing real-world implementation challenges, and assisting in the interpretation of feasibility metrics and qualitative findings.

In the post-study phase, the team will organize a dedicated outcome-sharing event to gather all participants and stakeholders. This event serves as a platform for knowledge translation, allowing the research team to present findings directly to the community while gathering final feedback on the protocol’s ecological validity. By involving stakeholders as active partners rather than passive subjects, this approach ensures the study design remains participant-centered and provides a refined foundation for future large-scale clinical trials.

### 3.7. Study timeline

The project has enrolled its first participant on November 18^th^ 2025, and enrollment is expected to be completed in May 2026. Recruitment will continue until the last participating dyad has been confirmed, and data collection is plan to be done before July 2026. The final results are expected to be presented in December 2026.

## 4. Expected outcomes and discussion

Based on the research objectives, this study anticipates the following three merits:

1. Improving the application of wearable devices for patients with BPSD: By evaluating the acceptability and feasibility of Geneactiv and Re-Timer devices, this study will provide an abundance of information to help optimize their design and usage, thereby increasing the willingness of patients with dementia and their caregivers to use these devices regularly in daily life.
2. Collection of preliminary data on clinical efficacy: The study will generate initial data on the effects of Re-Timer on BPSD and sleep problems of AD patients and the stress and sleep on their caregivers. These findings will help assess whether the device can improve the quality of life for patients and caregivers and provide direction and baseline information for future large-scale studies.
3. Exploring the impact of light and circadian rhythm on BPSD: By analyzing long-term activity patterns and light exposure data collected via Geneactiv, this study will explore the relationship between light, circadian rhythm, and changes in BPSD symptoms. This may offer clinically meaningful insights for developing new non-pharmacological interventions and improving the assessment and management of BPSD.

Overall, despite its smaller sample size, this pilot study will be the first dyadic study exploring the feasibility of applying two wearable devices concomitantly on AD patients with pre-defined BPSD and their caregiver. Under the context of global ageing, the burden of dementia has not only led to huge caregiver burden, but also heightened societal and healthcare costs. If proved feasible in rural Taiwan where both digital and health literacy and resources are limited, our study may be able to guide future research in designing more customized wearable-based dementia care models that ameliorate BPSD and reduce caregiver burden.

### 4.1. Trial registration

The trial has been pre-registered in ClinicalTrials.gov with identifying number: NCT07249918 (URL: https://clinicaltrials.gov/study/NCT07249918)

## Supporting information

S1 File Interview guide. (DOCX)

S2 File Original study protocol approved by CMUHREC. (PDF)

S3 File ENG_translated study protocol approved by CMUHREC. (PDF)

S4 File SPIRIT checklist. (DOCX)
