# Supplementary material for: Facilitating the measurement and treatment of Behavioral and Psychological Symptoms of Dementia (BPSD) and understanding caregiver burden using wearable devices in Rural Taiwan—Protocol for a dyadic feasibility pilot study
Source: PLoS One. 2026 May 18;21(5):e0342136. doi: 10.1371/journal.pone.0342136 (PMC13183198; doi:10.1371/journal.pone.0342136)
Supplement: S1 File — (DOCX) [file pone.0342136.s001.docx]

**Supplementary Materials**

**Interview guide for the semi-structured interview extended from T-QUEST**

**Research Rigor**

This study adopts Lincoln & Guba’s criteria (Lincoln 1985) to ensure rigor and credibility in qualitative research, including:

- **Credibility**: Equivalent to internal validity in quantitative research; ensures findings reflect participants’ experiences and cultural context. Strategies include continuous literature review, prolonged observation, full audio recording with verbatim transcription, non-verbal notes, reflective journaling, participant verification, and peer discussion.
- **Transferability**: Equivalent to external validity; ensures findings can apply to similar cases. Achieved through purposive sampling, clear inclusion criteria, and in-depth interviews encouraging authentic responses.
- **Dependability**: Equivalent to reliability; ensures consistency of findings with collected data. Strategies include fixed interview times, audio recording, reflective journaling, repeated verification with participants, and peer review.
- **Confirmability**: Equivalent to objectivity; ensures neutrality by preserving all recordings, transcripts, and analysis documents for audit. Analysis reviewed by two additional experts to enhance verifiability.

**Interview Guide Content**

1. How do you feel after using this device?
2. What changes have occurred in your daily life since using the device?
3. Do you feel the device has helped you or brought any changes? (Note: For caregiver, change can be associated with any alteration of the patient he/she is taking care of.)
4. How do you feel about the size and weight of the device?
5. How do you feel about adjusting or operating the device?
6. Do you have any concerns about using the device?
7. How comfortable do you feel when using the device?
8. Have you encountered any difficulties while using it?
9. Do you think this device is easy to use? Why?
10. What do you think are the advantages and disadvantages of using this device? (Note: For caregiver, advantages and disadvantages can be any alteration of the patient he/she is taking care of and the subsequent influence on the caregiving burden.)
11. Looking back, what has been the greatest benefit of using this device?
12. Do you have any additional comments or suggestions regarding this device?

**Reference**

Lincoln, Y. S., and Guba, E. G. . 1985. *Naturalistic inquiry.* . Newbury park, CA: Sage.
