# Supplementary material for: Facilitating the measurement and treatment of Behavioral and Psychological Symptoms of Dementia (BPSD) and understanding caregiver burden using wearable devices in Rural Taiwan—Protocol for a dyadic feasibility pilot study
Source: PLoS One. 2026 May 18;21(5):e0342136. doi: 10.1371/journal.pone.0342136 (PMC13183198; doi:10.1371/journal.pone.0342136)
Supplement: S2 File — (PDF) [file pone.0342136.s002.pdf]

113 年度中國醫藥大學北港附設醫院研究計畫申請書

一、計畫中文摘要：

以穿戴式裝置輔助評估與改善台灣偏鄉失智症精神行為症狀與照護壓力之前驅研究

請於五百字內就本計畫要點作一概述，並依本計畫性質自訂關鍵詞。

關鍵詞：穿戴式裝置、精神行為症狀、睡眠、照護壓力

2025 年台灣將成為超高齡社會，失智症患者亦持續上升，大多數患者會產生包含躁動、睡眠障礙等「精神行為症狀（Behavioral and Psychological Symptoms of Dementia, BPSD）」，其不但使病人生活品質惡化，也提高照護者壓力令其易產生憂鬱、睡眠障礙。

目前 BPSD 評估仰賴對照護者的問卷訪談，不但較為片面，且因照護者也常為認知退化的長輩，難以準確評估，進而影響治療計畫擬定。本人於英國進行的研究發現，重度失智之長輩仍能長期被動配戴如 Geneactiv 等研究級腕動儀，其評估不但較客觀、連續，所分析得到的新興變量（如睡眠穩定指數，Sleep Regularity Index）亦有助預測認知與身體功能。

此外，BPSD 治療過度仰賴藥物，但許多藥物並未取得適應症，且有研究顯示會增加失智長輩跌倒與死亡風險。能發出特定綠光的光學眼鏡 Re-Timer 已證實可安全地改善成人睡眠與情緒，若失智症長輩也能夠穿戴，可能可改善特定 BPSD，進而增進病人與照護者的生活品質。

過去台灣少有應用穿戴式裝置於失智症病人之研究，本研究之主要目標為了解「評估型」與「治療型」兩類的穿戴式裝置在台灣已有顯著 BPSD 的失智症病人以及其照護者之適用性。次要目標則為探索配戴 Re-Timer 在減緩睡眠與特定 BPSD 以及照護壓力上的初步療效，及比較 Geneactiv 和問卷所得到之評估差異，提供未來「電子生物指標」研究基礎。

二、計畫英文摘要：請於五百字內就本計畫要點作一概述，並依本計畫性質自訂關鍵詞。

Keywords：Wearable device, Behavioral and Psychological Symptoms of Dementia (BPSD), Sleep, Caregiver Burden

Taiwan is predicted to become a super-aged society, with people older than 65-year-old exceeding 20 percent. As the prevalence of Alzheimer's dementia (AD) increases with age, becoming a super-aged society means the number of AD patients is also going to increase. Most of the AD patients will experience at least one type of behavioral and psychological symptoms of dementia (PBSD). BPSD not only worsen the quality of life among the patients, they also increase caregiver burden and make the caregivers more prone to depression and sleep disturbances.

Questionnaires answered by caregivers are currently the standard tools for BPSD assessment and measurement. Although these questionnaires have been validated, they can only provide cross-sectional results. In a super-aged society where caregivers themselves are commonly also aged with declined memory and verbal function, these caregiver-rated questionnaires may also be biased and often imprecise. Based on my previous study in the UK, late-stage AD patients could still passively wear a research-grade actigraphy (Geneactiv) with good compliance. Device-based measurement can provide more objective and longitudinal information, and some of the innovative device-based variables (such as the 'Sleep Regularity Index, SRI') are also of predictive value for both cognitive and physical functionality.

In addition, BPSD are currently treated mostly with pharmacotherapy, despite the fact that most medications are not approved for BPSD, and many of them have been associated with increased risk of fall and mortality. Therapeutical wearable device, such as 'Re-Timer,' an eyewear emitting specific wavelength of green light, has been shown to improve sleep and mood symptoms in general adult population with good safety profile. If this device can be accepted by AD patients with BPSD and their caregivers, it might ameliorate their BPSD, and improve the quality of life in both the patients and their caregivers.

Very few studies had applied wearable device in dementia patients in Taiwan. Therefore, the primary outcome of this study is to establish the feasibility and acceptability of both measuring and interventional wearable device in AD patients with significant BPSD, and in their caregivers. The secondary outcomes of this studies include exploring the efficacy of Re-Timer in reducing specific BPSD and caregiver burden, as well as comparing the differences between questionnaire-based measurements and device-based measurements, to form a basis for future studies to develop 'digital biomarkers' in AD patients.

三、研究計畫之背景及目的：請詳述本研究計畫之背景、目的、重要性以及國內外有關本計畫之研究情況，重要參考文獻等。

研究背景:

台灣將於 2025 年進入老年人口占比達百分之二十以上之「超高齡社會」，失智症患者的人數不斷上升恐將帶來龐大的照護壓力(Cotton & Verghese, 2024)。根據統計，台灣的失智症長輩往往同時有多重慢性病，且使用大量醫療照護資源(Huang et al., 2024)。在照護上，失智症病人的精神行為症狀（Behavioral and Psychological Symptoms of Dementia, BPSD，或神經精神症狀，Neuropsychiatry Symptoms），尤其「憂鬱（depression）」、「躁動/攻擊（agitation/aggression）」、「淡漠（apathy）」與「夜間睡眠症狀（nocturnal sleep）」等，常比認知症狀耗費更多照護資源，也造成更大的照護者壓力(Feast et al., 2016)。

BPSD 的診斷與評估完全仰賴臨床訪談或問卷(Stella, 2013)。而由於產生 BPSD 的失智症病人本身多已無法確切記憶、表達自身症狀，這些問卷的施測對象多為病人的照護者(Cummings, 2020; Mao et al., 2015)，對於邁入超高齡社會、85%的照護者為非專業家庭照護者的台灣來說，許多照護者也無法確切描述病人的 BPSD(Cotton & Verghese, 2024)，進而影響醫療端評估與擬定後續的治療計畫，例如因為無法確知 BPSD 是否改善，因而長期處方可能增加病人跌倒風險的鎮定安眠藥，與不得已使用可能增加病人心血管疾病與死亡風險、但實際上並非仿單所許可（off-label）的抗精神病藥等(Huang et al., 2024)。

過度依賴風險較高卻非適應症的藥物也反映出目前對於 BPSD 產生機轉尚不明瞭。部分研究顯示造成阿茲海默型失智症的類澱粉沉積與 Tau 蛋白也可能造成 BPSD(Ehrenberg et al., 2018)，但近年有更多研究認為 BPSD 的產生可能並非透過單一機轉，包括生理疾病、知覺（尤其聽力與視力）退化以及環境條件（如環境中的光線）等也可能誘發或者惡化 BPSD(Corbett et al., 2013; Guu et al., 2022)，因此妥善評估 BPSD 的促發與惡化因子應優先於給予治療(Kales et al., 2015)。

穿戴式裝置若能被失智長輩接受並且規則配戴，將可能在評估端與治療端皆帶來突破性的改變，亦有助於了解失智症的進程與 BPSD 的產生機轉。在評估端，本人於英國的研究近期發現，重度失智且合併有顯著躁動攻擊症狀的失智症患者也能妥善配戴研究等級之腕動儀 Geneactiv 連續四周以上，且 BPSD 的症狀與嚴重度並不會影響其對穿戴式裝置的接受度與配戴率(Guu et al., 2024)。類似 Geneactiv 等研究等級穿戴式裝置不僅能透過經大規模驗證的開放式演算法評估配戴者的睡眠行為(van Hees et al., 2015)，更能進一步分析此類數據的長期動態變化，例如與心血管疾病之發生高度負相關、而與失智症發生有 U 行相關的「睡眠穩定指數（Sleep Regularity Index）」、輔助評估躁動與睡眠障礙等

BPSD、甚至了解光線對於 BPSD 的影響(Guu, 2024)。

在治療上，過去已有研究失智症病人產生 BPSD 的可能原因之一為接收到的環境光線不足，因此尤其容易出現在缺乏室外活動機會的養護機構中(Guu et al., 2022)。此外雖然有研究發現特定光譜之環境光線或者光照治療可能有助於改善諸如憂鬱、夜間睡眠紊亂等 BPSD，但療效並不一致(Hjetland et al., 2021; Hjetland et al., 2020; Kolberg et al., 2021)，其中可能的原因包括環境光線距離失智症病人的眼睛較遠，而失智症病人對於光線的接收能力亦已退化，造成光線的實際療效難以顯現(Guu et al., 2022)。

“Re-Timer” (http://re-timer.com/)為一眼鏡型、可發出波長 500nm 藍綠光的穿戴式裝置，有初步研究發現其對於成人憂鬱、日夜節律調節以及睡眠症狀可能有療效(Corbett, 2013; Lovato & Lack, 2016; Zalta et al., 2019)，另也有前驅研究發現，有輕微憂鬱與睡眠障礙的老年長者亦能規則配戴此裝置每天三十分鐘並持續兩周，且睡眠節律可能受此裝置調節(Leggett et al., 2018)。若失智長輩能規則配戴此類裝置更長的時間，或許有機會以此非藥物的方式改善其 BPSD，減少失智症病人暴露在過多高風險藥物的機會；而若期照護者也能同步配戴，或許能改善其照護壓力。

研究目的:

1. 主要目的（Primary outcome）了解兩種不同的穿戴式裝置：Geneactiv 以及 Re-Timer，在已有顯著 BPSD 的失智症患者以及其照護者之接受度（Acceptability）與於生活中長時間配戴的可行性（Feasibility）；
2. 次要目的（Secondary outcomes）獲得此裝置在 BPSD 以及睡眠症狀上的初步療效資訊，以協助評估計算後續研究所須之研究樣本大小以及研究時間，以及初步了解光線及日夜節律與 BPSD 之發生與變化的關係。

研究重要性:

1. 確認已有顯著 BPSD 之台灣偏鄉失智症病人以及其照護者對於穿戴式裝置之接受度及適用性，取得台灣本土代表性資料(Guu et al., 2023)，將有助於進入超高齡社會的台灣建立以穿戴式裝置評估、治療 BPSD 的新照護模式。
2. 為區分日間與夜間 BPSD 提供更進一步的客觀證據(Cummings et al., 2024)，協助臨床照護者更全面性的考慮環境因子與生活型態對人類行為造成的影響，以提供更客製化的治療計畫。

參考文獻:

Buysse, D. J., Reynolds, C. F., 3rd, Monk, T. H., Berman, S. R., & Kupfer, D. J. (1989). The Pittsburgh Sleep Quality Index: a new instrument for psychiatric practice and research. *Psychiatry Res*, *28*(2), 193-213. https://doi.org/10.1016/0165-1781(89)90047-4

Carrillo, M. C., Dean, R. A., Nicolas, F., Miller, D. S., Berman, R., Khachaturian, Z., Bain, L. J., Schindler, R., & Knopman, D. (2013). Revisiting the framework of the National Institute on Aging-Alzheimer's Association diagnostic criteria. *Alzheimer's & Dementia*, *9*(5), 594-601. https://doi.org/https://doi.org/10.1016/j.jalz.2013.05.1762

Chou, K. R., Jiann-Chyun, L., & Chu, H. (2002). The reliability and validity of the Chinese version of the caregiver burden inventory. *Nurs Res*, *51*(5), 324-331. https://doi.org/10.1097/00006199-200209000-00009

Corbett, A., Nunez, K., & Thomas, A. (2013). Coping with dementia in care homes. *Maturitas*, *76*(1), 3-4. https://doi.org/10.1016/j.maturitas.2013.06.002

Corbett, M. A. (2013). A potential aid to circadian adaptation: re-timer. *Aviat Space Environ Med*, *84*(10), 1113-1114. https://doi.org/10.3357/asem.3827.2013

Cotton, K., & Verghese, J. (2024). Dementia in Taiwan. *Archives of Gerontology and Geriatrics*, *121*, 105415. https://doi.org/https://doi.org/10.1016/j.archger.2024.105415

Cummings, J. (2020). The Neuropsychiatric Inventory: Development and Applications. *J Geriatr Psychiatry Neurol*, *33*(2), 73-84. https://doi.org/10.1177/0891988719882102

Cummings, J., Sano, M., Auer, S., Bergh, S., Fischer, C. E., Gerritsen, D., Grossberg, G., Ismail, Z., Lanctôt, K., Lapid, M. I., Mintzer, J., Palm, R., Rosenberg, P. B., Splaine, M., Zhong, K., & Zhu, C. W. (2024). Reduction and prevention of agitation in persons with neurocognitive disorders: an international psychogeriatric association consensus algorithm. *Int Psychogeriatr*, *36*(4), 251-262. https://doi.org/10.1017/s104161022200103x

Ehrenberg, A. J., Suemoto, C. K., Franca Resende, E. P., Petersen, C., Leite, R. E. P., Rodriguez, R. D., Ferretti-Rebustini, R. E. L., You, M., Oh, J., Nitrini, R., Pasqualucci, C. A., Jacob-Filho, W., Kramer, J. H., Gatchel, J. R., & Grinberg, L. T. (2018). Neuropathologic Correlates of Psychiatric Symptoms in Alzheimer's Disease. *J Alzheimers Dis*, *66*(1), 115-126. https://doi.org/10.3233/JAD-180688

Farina, N., Sherlock, G., Thomas, S., Lowry, R. G., & Banerjee, S. (2019). Acceptability and feasibility of wearing activity monitors in community-dwelling older adults with dementia. *Int J Geriatr Psychiatry*, *34*(4), 617-624. https://doi.org/10.1002/gps.5064

Feast, A., Moniz-Cook, E., Stoner, C., Charlesworth, G., & Orrell, M. (2016). A systematic review of the relationship between behavioral and psychological symptoms (BPSD) and caregiver well-being. *Int Psychogeriatr*, *28*(11), 1761-1774.

https://doi.org/10.1017/s1041610216000922

Guu, T.-W., Brem, A.-K., Albertyn, C. P., Kandangwa, P., Aarsland, D., & ffytche, D. (2024). Wrist-worn actigraphy in agitated late-stage dementia patients: A feasibility study on digital inclusion. *Alzheimer's & Dementia*, *20*(5), 3211-3218. https://doi.org/https://doi.org/10.1002/alz.13772

Guu, T. W. (2024). *STAND-S study - explore sleep, circadian rhythm, light and neuropsychiatry symptoms in dementia with wearable devises* King's College London]. Institute of Psychiatry, Psychology and Neuroscience. https://kclpure.kcl.ac.uk/portal/en/studentTheses/stand-s-study-explore-sleep-circadian-rhythm-light-and-neuropsych

Guu, T. W., Aarsland, D., & Ffytche, D. (2022). Light, sleep-wake rhythm, and behavioural and psychological symptoms of dementia in care home patients: Revisiting the sundowning syndrome. *Int J Geriatr Psychiatry*, *37*(5). https://doi.org/10.1002/gps.5712

Guu, T. W., Muurling, M., Khan, Z., Kalafatis, C., Aarsland, D., Ffytche, D., & Brem, A. K. (2023). Wearable devices: underrepresentation in the ageing society. *Lancet Digit Health*, *5*(6), e336-e337. https://doi.org/10.1016/s2589-7500(23)00069-9

Hjetland, G. J., Kolberg, E., Pallesen, S., Thun, E., Nordhus, I. H., Bjorvatn, B., & Flo-Groeneboom, E. (2021). Ambient bright light treatment improved proxy-rated sleep but not sleep measured by actigraphy in nursing home patients with dementia: a placebo-controlled randomised trial. *BMC Geriatr*, *21*(1), 312. https://doi.org/10.1186/s12877-021-02236-4

Hjetland, G. J., Pallesen, S., Thun, E., Kolberg, E., Nordhus, I. H., & Flo, E. (2020). Light interventions and sleep, circadian, behavioral, and psychological disturbances in dementia: A systematic review of methods and outcomes. *Sleep Med Rev*, *52*, 101310. https://doi.org/10.1016/j.smrv.2020.101310

Huang, S.-T., Loh, C.-H., Lin, C.-H., Hsiao, F.-Y., & Chen, L.-K. (2024). Trends in dementia incidence and mortality, and dynamic changes in comorbidity and healthcare utilization from 2004 to 2017: A Taiwan national cohort study. *Archives of Gerontology and Geriatrics*, *121*, 105330. https://doi.org/https://doi.org/10.1016/j.archger.2024.105330

Jack, C. R., Jr., Albert, M. S., Knopman, D. S., McKhann, G. M., Sperling, R. A., Carrillo, M. C., Thies, B., & Phelps, C. H. (2011). Introduction to the recommendations from the National Institute on Aging-Alzheimer's Association workgroups on diagnostic guidelines for Alzheimer's disease. *Alzheimers Dement*, *7*(3), 257-262. https://doi.org/10.1016/j.jalz.2011.03.004

Kales, H. C., Gitlin, L. N., & Lyketsos, C. G. (2015). Assessment and management of behavioral and psychological symptoms of dementia. *BMJ*, *350*, h369. https://doi.org/10.1136/bmj.h369

Kolberg, E., Hjetland, G. J., Thun, E., Pallesen, S., Nordhus, I. H., Husebo, B. S., & Flo-Groeneboom, E. (2021). The effects of bright light treatment on affective symptoms in people with dementia: a 24-week cluster randomized controlled trial. *BMC Psychiatry*, *21*(1), 377. https://doi.org/10.1186/s12888-021-03376-y

Leggett, A. N., Conroy, D. A., Blow, F. C., & Kales, H. C. (2018). Bright Light as a Preventive Intervention for Depression in Late-Life: A Pilot Study on Feasibility, Acceptability, and Symptom Improvement. *Am J Geriatr Psychiatry*, *26*(5), 598-602. https://doi.org/10.1016/j.jagp.2017.11.007

Lovato, N., & Lack, L. (2016). Circadian phase delay using the newly developed re-timer portable light device. *Sleep and Biological Rhythms*, *14*(2), 157-164. https://doi.org/10.1007/s41105-015-0034-6

Lunsford-Avery, J. R., Engelhard, M. M., Navar, A. M., & Kollins, S. H. (2018). Validation of the Sleep Regularity Index in Older Adults and Associations with Cardiometabolic Risk. *Sci Rep*, *8*(1), 14158. https://doi.org/10.1038/s41598-018-32402-5

Lyketsos, C. G., Lopez, O., Jones, B., Fitzpatrick, A. L., Breitner, J., & DeKosky, S. (2002). Prevalence of neuropsychiatric symptoms in dementia and mild cognitive impairment: results from the cardiovascular health study. *JAMA*, *288*(12), 1475-1483. https://doi.org/10.1001/jama.288.12.1475

Mao, H. F., Chen, W. Y., Yao, G., Huang, S. L., Lin, C. C., & Huang, W. N. (2010). Cross-cultural adaptation and validation of the Quebec User Evaluation of Satisfaction with Assistive Technology (QUEST 2.0): the development of the Taiwanese version. *Clin Rehabil*, *24*(5), 412-421. https://doi.org/10.1177/0269215509347438

Mao, H. F., Kuo, C. A., Huang, W. N., Cummings, J. L., & Hwang, T. J. (2015). Values of the Minimal Clinically Important Difference for the Neuropsychiatric Inventory Questionnaire in Individuals with Dementia. *J Am Geriatr Soc*, *63*(7), 1448-1452. https://doi.org/10.1111/jgs.13473

Stella, F. (2013). Assessment of neuropsychiatric symptoms in dementia: toward improving accuracy. *Dement Neuropsychol*, *7*(3), 244-251. https://doi.org/10.1590/S1980-57642013DN70300003

van Hees, V. T., Sabia, S., Anderson, K. N., Denton, S. J., Oliver, J., Catt, M., Abell, J. G., Kivimaki, M., Trenell, M. I., & Singh-Manoux, A. (2015). A Novel, Open Access Method to Assess Sleep Duration Using a Wrist-Worn Accelerometer. *PLoS One*, *10*(11), e0142533. https://doi.org/10.1371/journal.pone.0142533

Zalta, A. K., Bravo, K., Valdespino-Hayden, Z., Pollack, M. H., & Burgess, H. J. (2019). A placebo-controlled pilot study of a wearable morning bright light treatment for probable PTSD. *Depress Anxiety*, *36*(7), 617-624. https://doi.org/10.1002/da.22897

四、研究方法及進行步驟：

1.請細述本計畫採用之研究方法與原因。
2.預計可能遭遇之困難及解決途徑。
3.重要儀器之配合使用情形。
4.一年期以上之計畫，請分年列述。
5.本計畫如為整合型計畫，請就以上各點分別說明與其他子計畫之相關性。

研究方法概述：

本研究以北港附設醫院身心科、神經內科門診，以及附屬之元長失智症日照中心、五個失智症據點中，已有顯著睡眠、躁動、淡漠或憂鬱等四種精神行為症狀至少其中之一的失智症病人，以及其主要照護者為收案對象，經由照護團隊（包含醫師、失智症個案管理師或護理師）向病人與主要照護者詢問是否有意願加入此研究，有意加入研究之病人及照護者再由研究主持人加以說明並進行對應的介入措施。

本研究設計上為單臂配對前驅研究（single arm dyadic pilot study）。病人與照護者皆須配戴四週、每天至少三十分鐘之 Re-Timer 光學眼鏡，並於開始配戴 Re-Timer 前、後兩周，以及配戴 Re-timer 之四週期間，配戴 Geneactiv 腕動儀共八週。

研究對象：

1. 收案對象：預計收案 10 位病患與 10 位患者之主要照護者。
2. 收案標準：

（1）病患：

（a）臨床診斷患有極可能為阿茲海默型失智症（Probable AD）者，以及CDR 0.5分因AD造成之輕度知能障礙（Mild Cognitive Impairment due to AD)患者(Carrillo et al., 2013; Jack et al., 2011)。

（b）合併有「顯著」之「憂鬱」、「躁動」、「淡漠」、「夜間睡眠障礙」等四種BPSD至少一種之病人。其中「顯著」之定義為Neuropsychiatry Inventory（NPI）單項症狀分數四分以上（即嚴重度x發生頻率的乘積四分以上）(Lyketsos et al., 2002)，或Neuropsychiatry Inventory Questionnaire（NPI-Q）嚴重度分數兩分以上；若主要症狀包含睡眠問題，則可為PSQI總分五分以上者(Buysse et al., 1989)。

（c）維持於目前之臨床場域兩週以上（如在社區收案者須已在該住處居住兩週以上，在日間照護中心收案者須已在日照中心規則參與活動兩週以上）

（d）若目前正以藥物或非藥物方式治療BPSD者，治療劑量、頻次需在參與本臨床試驗前已穩定兩週以上。

（e）不符合排除條件，同意參與並簽署受試同意書者。若臨床判定心智功能已退化至已無法自行簽署受試者同意書者，須有法律代理人同意並協助簽署受試同意書。

（2）主要照護者：

（a）受試患者之成年主要照護者。

（b）若目前正以藥物或非藥物方式處遇照護壓力者，處遇之劑量、頻次需在參與本臨床試驗前已穩定兩週以上。

（c）不符合排除條件且擔任患者之主要照顧者，並簽署受試者同意書者。

3. 排除條件：

（1）可能因此研究受到傷害的族群，包括有視網膜疾患、目前正在使用光敏性（photosensitizing）藥物、四周內曾經接受眼部手術尚未復原、以及有其他身心狀況不適合接受藍綠光線照射者（如癲癇）。

（2）身心狀況不穩定經臨床評估不適合者，例如目前處於急性譫妄、患有呼吸道感染症（包含 COVID）等。

研究設備：

1. Geneactiv 腕動儀之外觀為手錶造型，表面並有一個感光元件可偵測所處環境中的光線強度。其為少數已於成人及老人族群驗證過之研究用腕動儀。雖然尚無於本土失智症族群進行之相關研究，但近年包含本人於英國進行之研究顯示，居住在社區之輕度失智症患者以及養護機構之重度失智症患者皆能接受並規則配戴此裝置(Farina et al., 2019; Guu et al., 2024)。

   研究人員將在研究開始時，協助受試者配戴此腕動儀，因本人過去研究發現失智症患者配戴此類裝置仍可能因為裝置的美觀與設計影響其配戴意願(Guu et al., 2024)，因此研究中將提供原廠認定適用之替換表帶供受試者選擇。確認配戴時無不適感受（不會過緊或過鬆）後，研究人員將指導個案於研究過程中若感到不適，隨時可以短暫取下後再嘗試重新配戴或者不再配戴，另也將提醒個案避免以衣物遮蔽表面之感光元件。

2. Re-Timer 生理時鐘調節器為一眼鏡型之光學調控裝置，其可散發出波長 500nm 之藍綠色可見光，無紫外線，已經衛福部驗證不以醫材列管。受試個案使用此眼鏡之能量功率（照度與能量分別為 506 Lux Im/m2，230 μW/cm2)已於數個早期研究驗證其於一般成人、有特定精神疾病之成人、以及老年族群之適用性與安全性(Corbett, 2013; Leggett et al., 2018; Lovato & Lack, 2016; Zalta et al.,

2019)。研究人員將指導並確認受試者正確配戴此裝置使藍綠光之照射範圍包含眼睛瞳孔之位置，個案於研究過程中若感到不適，隨時可以短暫取下後再嘗試重新配戴或者不再配戴。

研究執行與介入方式：

1. 所有參與者於研究開始的第一天起配戴 Geneactiv 腕動儀，配戴至第八週結束（第 56 天），共配戴八週。
2. 門診之失智症病人以及所有願意參與配戴 Re-Timer 的主要照護者將於第十五天起配戴 Re-Timer 至第四十二天，共配戴四周，每天配戴至少三十分鐘，至多六十分鐘，並記錄配戴的時段。日照中心與失智症據點之失智症病人將於第十五天起配戴 Re-Timer 至第四十二天，共配戴四周，每天配戴至少三十分鐘，至多六十分鐘，並記錄配戴的時段，可以選擇僅在據點或者日照中心參與活動時，由人員協助配戴，或者將 Re-Timer 帶回家配戴。
3. 配戴裝置時若有任何不適之症狀，受試者除可以立刻暫停配戴之外，研究人員亦將指導、協助個案通報並紀錄不適之症狀或反應，由研究人員轉介相關醫療服務，必要時亦可退出研究。

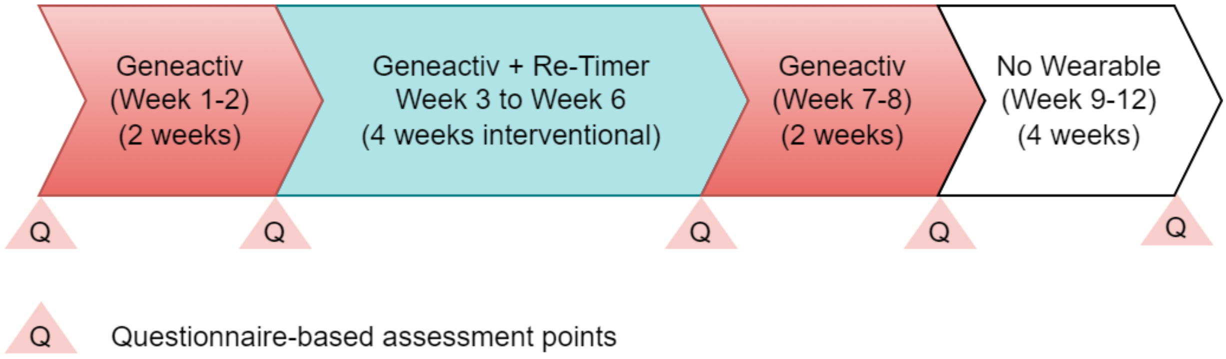

圖一、研究流程示意圖：研究為期十二週，受試者共需配戴至多八週之 Geneactiv 腕動儀，至多四週之 Re-Timer，並於試驗開始第一天、第十五天、第四十二天、第五十六天與第八十四天接受五次問卷評估（各時間點評估之項目如「評估項目與方式」)。

評估項目與方式：

1. 基本資料蒐集：

（1） 失智症病人：出生年月日、性別、婚姻狀態、教育程度、失智症（或 MCI）診斷時間、目前認知功能分級（可為六個月內檢測之臨床失智量表（Clinical Dementia Rating, CDR)、認知功能障礙篩檢量表（Cognitive Abilities Screening Instrument, CASI）或簡易心智量表（Mini Mental State

Examination,MMSE）之分數)，若近期可能有顯著認知功能變化，得重新施測並以最近一次施測結果為準)、開始出現 BPSD 之時間、目前失智症相關用藥、吸菸狀況、飲酒狀況、內外科與精神科疾病史與目前治療。

（2） 照護者：出生年月日、性別、婚姻狀態、教育程度、吸菸狀況、飲酒狀況、內外科與精神科疾病史與目前治療狀況。若照護者亦為認知功能可能已退化之高齡長輩，亦將收集目前認知功能分級(可為六個月內檢測之 CDR、CASI 或 MMSE 之分數，若近期可能有顯著認知功能變化，得重新施測並以最近一次施測結果為準)。

2. 問卷評估：

（1） 匹茲堡睡眠量表（Pittsburgh Sleep Quality Index, PSQI）(Buysse et al., 1989)：共有七大面向與睡眠相關之問題，每題的分數介於零分(無此症狀)至三分之間。於試驗開始第一天、第十五天、第四十二天、第五十六天與第八十四天共評估五次，分別施測於照護者及失智症患者。若失智症患者經臨床判斷可自行填答，則由此患者自行填答，否則可由照護者代答。

（2） 中文版簡短神經精神量表（Neuropsychiatry Inventory Questionnaire, NPI-Q）(Mao et al., 2015)：共評估十二項行為症狀，每項症狀依序評估「是否有此症狀」、「嚴重度(對個案造成之影響，一至三分)」與「干擾程度(對照護者造成之影響，零至五分)」。於試驗開始第一天、第十五天、第四十二天、第五十六天與第八十四天共評估五次，每一位受試個案於試驗中由固定一位主要照護者評估回答。

（3） 中文版照顧者負擔量表（Caregiver Burden Inventory, CBI）(Chou et al., 2002)：評估五大面向共 24 題(每題 0-4 分)，總分越高代表負荷越重。於試驗開始第一天、第四十二天與第八十四天共評估三次，每一位受試個案於試驗中由固定一位主要照護者評估回答。

（4） 台灣版魁北克輔具使用者滿意度量表（Taiwanese Version of the Quebec User Evaluation of Satisfaction with Assistive Technology, T-QUEST）(Mao et al., 2010)：用於評估受試者對穿戴式裝置及其相關服務的滿意狀況，共包含 13 項題目，每一題之分數從「1 分－極不滿意」至「5 分－極滿意」。分別施測於照護者及失智症患者，於第四十二天、第五十六天分別評估對 Re-Timer、Geneactiv 之滿意度。若失智症患者經臨床判斷無法填答，則僅由有配戴穿戴式裝置之照護者回答。

3. 半結構性訪談：將延伸 T-QUEST 問卷問題，開放性地訪談以了解失智症受試者和照護者對兩種裝置的接受度、使用便利性、舒適度以及可能帶來的困難。每次訪談將持續約 30-60 分鐘，訪談地點將選擇受試者和照護者感到舒適的環境，以確保資料收集過程的自然性和可靠性。資料收集完成後，訪談錄音將被轉錄為文字稿，並使用主題分析法進行分析。以探索其在參與臨床試驗後對於以穿戴式裝置評估、治療精神疾病的想法。

資料分析與呈現：

1. 所有統計資料將以 R studio 與 R 進行分析，基本資料將以描述性統計根據數據之性質計算平均值、百分比與標準差等。

2. **主要目的（Primary outcome）評估與統計分析：**將綜合量性與質性分析之方式完整評估兩種穿戴式裝置的接受度與適用性。

（1） 量性分析上，將使用經驗證過之開放性演算軟體 GGIR 計算每位個案的腕動儀實際配戴時間(van Hees et al., 2015)，每一位個案的 Geneactiv 配戴率計算方式為（實際配戴時間／可配戴時間）；Re-Timer 之配戴時間由配戴者自行記錄（失智症病人的配戴時間若可能無法準確記憶則由期照護者協助記錄），加總每日配戴的時間後，每一位個案的 Re-Timer 配戴率計算方式為（總配戴時間／可配戴時間）。

接著將透過計算 T-QUEST 總分，以了解受試者對於兩種穿戴式裝置各面向之滿意度。失智症與照護者對於兩種裝置的接受度與適用性差異將以獨立 t 檢定分析。

（2） 質性分析上，首先，研究團隊將首先通讀所有訪談稿，進行初步編碼，以識別與研究問題相關的重點主題。接著，研究人員將根據初步編碼結果進行細緻的主題分類，將不同的主題加以整合，並找出受試者和照護者之間的共通點和差異。過程中，研究團隊將持續進行比較，以確保編碼的一致性與分析的準確性。

最後，我們將進行三角驗證，透過不同研究者的獨立編碼與比較，確保資料分析的信度和研究結果的可信性。我們會考量受試者和照護者的背景差異（如年齡、文化、疾病進程等）對其看法和經驗的影響，以進一步探討穿戴式裝置在評估和治療精神疾病方面的潛在應用與挑戰。

3. **次要目的（Secondary outcome）評估與統計分析：**

在此研究中，我們的次要目的是探索 Re-Timer 對於失智症患者的 BPSD 及患者與

照護者在睡眠症狀的初步療效。我們將從穿戴式裝置所蒐集的連續數據中，以 GGIR 提取關鍵變項，包括活動量、睡眠便量（包含入睡時間、清醒次數、起床時間、睡眠時間總長度、睡眠穩定指數（Sleep Regularity Index）(Lunsford-Avery et al., 2018)等），以及光照暴露量等。這些數據將與 NPI-Q 以及 PSQI 進行時間序列的配對，初步探索光線暴露、日夜節律與 BPSD 變化的關聯性。

我們將採用混合效應模型（Mixed Effect Model）來分析這些縱向（Longitudinal）數據，這樣可以考慮重複測量以及個體之間的異質性。研究將通過設計對照前後的數據比較，評估穿戴式裝置是否對 BPSD 與睡眠症狀有顯著改善。分析將納入多重共變數，諸如年齡、疾病嚴重程度、共病情況等，以確保對潛在混淆因子的控制。對於光線與日夜節律的影響，我們會特別探討是否存在劑量效應，即不同光照強度和暴露時間對 BPSD 和睡眠症狀的影響是否呈現劑量反應。

最後，我們將基於這些初步結果進行功效分析，計算後續研究所需的樣本大小和研究時間。我們將透過計算 Cohen's d 值來估算效果量（effect size）大小，並根據研究假設的預期效應大小、顯著性水平和統計功效，來推算後續研究的樣本量和試驗設計。

公眾與病人參與（Public-Patient Involvement and Engagement, PPIE）：

在這項研究中，為確保研究設計和結果能充分反映患者和照護者的需求與觀點，促進公眾及患者參與，將設計並納入以下 PPIE 活動：

1. 諮詢團體與會議：我們預計透過此研究邀請失智症患者、照護者、相關的非營利組織代表及醫療專業人員成立諮詢團體（Advisory Group）並召開諮詢會議。會議的目為研究早期收集對穿戴式裝置使用的初步反饋，了解患者和照護者對裝置在日常生活中的可接受性與潛在挑戰。此外，我們將探討受試者對光照暴露（包含穿戴式裝置產生之光線與環境光線）和日夜節律調控的意見，確保這些措施對患者和照護者來說是實際可行且有意義的。通過這些諮詢，我們將根據他們的意見對未來研究設計進行優化，確保臨床試驗的設計符合患者和照護者的需求。
2. 研究結果解讀與知識傳播活動：在研究結束後，我們將組織患者和照護者參與研究結果的解讀會議，讓他們有機會了解並解釋研究數據。這不僅能提升研究結果的透明度，也能讓患者和照護者提供他們對結果的理解和解釋，尤其是穿戴式裝置對日常生活和 BPSD 症狀處遇的實際影響。此外，研究期間將舉辦公眾教育活動，將研究結果以簡單易懂的方式向更廣泛的患者群體及其家屬傳遞，促進研究成果的應用，並鼓勵未來更多的患者參與此類研究。

五、預期完成之工作項目及具體成果：

1. 請列述執行期限內預期完成之工作項目。
2. 對於學術研究、國家發展及其他應用方面預期之貢獻。
3. 對於參與之工作人員，預期可獲之訓練。
4. 一年期以上之計畫，請分年列述。
5. 本計畫如為整合型計畫之子計畫，請就以上各點分別說明與其他子計畫之相關性。

預期效益

根據上述研究目的，本研究有以下三項預期效益：

1. 改善穿戴式裝置在 BPSD 患者中的應用：透過評估 Geneactiv 與 Re-Timer 裝置的接受度和可行性，本研究可以幫助優化這些裝置的設計與應用，從而提升失智症患者及其照護者在日常生活中長時間使用這些裝置的意願。

2. 初步療效資料的收集：本研究將獲得 Re-Timer 對 BPSD 和睡眠問題的初步療效數據，這將有助於評估其是否能改善患者與照護者的生活，並為後續更大規模的研究提供方向和基礎數據。

3. 光線與日夜節律對 BPSD 影響：透過分析 Geneactiv 獲得的動態、長期活動量與光線暴露量資訊，本研究將初步探索光線和日夜節律對 BPSD 症狀變化的關聯，這可能為新的非藥物處遇方式提供具有臨床意義的訊息，有助於改善 BPSD 的評估與治療方式。

六、預定進度甘梯圖（Gantt Chart）：

1.本表作為進度控制評估之依據。

2.工作項目：請視計畫性質及需要自行訂定。預定進度以粗線標示其起迄月份，每月分三旬，如因農業或其他受季節性限制之計畫必須配合一定之月份者，請在(月次)欄下註明實際月份，以利審查。

3.預定進度累計百分比：係為配合追蹤考核作業所需，請視工作性質就以下因素擇一估計訂定：(1)工作天數，(2)經費之分配， (3)工作之比重， (4)擬達成目標之具體數字。

4.一年期以上之計畫，請分年列表。

5.本計畫如為整合型計畫之子計畫，請就以上各點分別說明與其他子計畫之相關性。

月 次 / 工作項目	第 1-2 月	第 3-4 月	第 5-6 月	第 7-8 月	第 9-10 月	第 11-12 月	第 13-14 月	第 15-16 月	第 17-18 月	第 19-20 月	第 21-22 月	第 23-24 月	備 註
研究設備布建與測試	......	......											
人體試驗與行政核可	......	......	...										
研究公告與收案宣傳				......	......	......	......						
研究收案				......	......	......	......	......					
質性訪談									......	......			
研究資料清理與分析									......	......			
研究論文撰寫									......	......	......	......	
公眾與病人參與活動											......	......	
預定進度累計百分比	5	10	15	20	30	40	50	60	70	80	90	100	
