# Supplementary material for: Facilitating the measurement and treatment of Behavioral and Psychological Symptoms of Dementia (BPSD) and understanding caregiver burden using wearable devices in Rural Taiwan—Protocol for a dyadic feasibility pilot study
Source: PLoS One. 2026 May 18;21(5):e0342136. doi: 10.1371/journal.pone.0342136 (PMC13183198; doi:10.1371/journal.pone.0342136)
Supplement: S3 File — (PDF) [file pone.0342136.s003.pdf]

Application Form for the 113th-Year Research Project of China Medical University Beigang Hospital

A. Chinese Abstract of the Project:

Facilitating the Measurement and Treatment of Behavioral and Psychological Symptoms of Dementia (BPSD) and Understanding Caregiver Burden Using Wearable Devices in Rural Taiwan – Protocol for a Dyadic Feasibility Pilot Study

Please provide a summary of the key points of this project within 500 words, and define keywords according to the nature of the project.

關鍵詞：穿戴式裝置、精神行為症狀、睡眠、照護壓力

2025 年台灣將成為超高齡社會，失智症患者亦持續上升，大多數患者會產生包含躁動、睡眠障礙等「精神行為症狀（Behavioral and Psychological Symptoms of Dementia, BPSD）」，其不但使病人生活品質惡化，也提高照護者壓力令其易產生憂鬱、睡眠障礙。

目前 BPSD 評估仰賴對照護者的問卷訪談，不但較為片面，且因照護者也常為認知退化的長輩，難以準確評估，進而影響治療計畫擬定。本人於英國進行的研究發現，重度失智之長輩仍能長期被動配戴如 Geneactiv 等研究級腕動儀，其評估不但較客觀、連續，所分析得到的新興變量（如睡眠穩定指數，Sleep Regularity Index）亦有助預測認知與身體功能。

此外，BPSD 治療過度仰賴藥物，但許多藥物並未取得適應症，且有研究顯示會增加失智長輩跌倒與死亡風險。能發出特定綠光的光學眼鏡 Re-Timer 已證實可安全地改善成人睡眠與情緒，若失智症長輩也能夠穿戴，可能可改善特定 BPSD，進而增進病人與照護者的生活品質。

過去台灣少有應用穿戴式裝置於失智症病人之研究，本研究之主要目標為了解「評估型」與「治療型」兩類的穿戴式裝置在台灣已有顯著 BPSD 的失智症病人以及其照護者之適用性。次要目標則為探索配戴 Re-Timer 在減緩睡眠與特定 BPSD 以及照護壓力上的初步療效，及比較 Geneactiv 和問卷所得到之評估差異，提供未來「電子生物指標」研究基礎。

B. English Abstract of the Project:

Please provide a summary of the key points of this project within 500 words, and define keywords according to the nature of the project.

Keywords：Wearable device, Behavioral and Psychological Symptoms of Dementia (BPSD), Sleep, Caregiver Burden

Taiwan is predicted to become a super-aged society, with people older than 65-year-old exceeding 20 percent. As the prevalence of Alzheimer's dementia (AD) increases with age, becoming a super-aged society means the number of AD patients is also going to increase. Most of the AD patients will experience at least one type of behavioral and psychological symptoms of dementia (PBSD). BPSD not only worsen the quality of life among the patients, they also increase caregiver burden and make the caregivers more prone to depression and sleep disturbances.

Questionnaires answered by caregivers are currently the standard tools for BPSD assessment and measurement. Although these questionnaires have been validated, they can only provide cross-sectional results. In a super-aged society where caregivers themselves are commonly also aged with declined memory and verbal function, these caregiver-rated questionnaires may also be biased and often imprecise. Based on my previous study in the UK, late-stage AD patients could still passively wear a research-grade actigraphy (Geneactiv) with good compliance. Device-based measurement can provide more objective and longitudinal information, and some of the innovative device-based variables (such as the 'Sleep Regularity Index, SRI') are also of predictive value for both cognitive and physical functionality.

In addition, BPSD are currently treated mostly with pharmacotherapy, despite the fact that most medications are not approved for BPSD, and many of them have been associated with increased risk of fall and mortality. Therapeutical wearable device, such as 'Re-Timer,' an eyewear emitting specific wavelength of green light, has been shown to improve sleep and mood symptoms in general adult population with good safety profile. If this device can be accepted by AD patients with BPSD and their caregivers, it might ameliorate their BPSD, and improve the quality of life in both the patients and their caregivers.

Very few studies had applied wearable device in dementia patients in Taiwan. Therefore, the primary outcome of this study is to establish the feasibility and acceptability of both measuring and interventional wearable device in AD patients with significant BPSD, and in their caregivers. The secondary outcomes of this studies include exploring the efficacy of Re-Timer in reducing specific BPSD and caregiver burden, as well as comparing the differences between questionnaire-based measurements and device-based measurements, to form a basis for future studies to develop 'digital biomarkers' in AD patients.

C. Background and Aims of the Research Project:

Please describe in detail the background, objectives, and significance of this study, as well as relevant domestic and international research and key references related to this project.

Background:

Taiwan will enter a “super-aged society” in 2025, defined as having more than 20% of its population aged 65 and older. The continually rising number of people with dementia is expected to generate a substantial caregiving burden (Cotton & Verghese, 2024). Statistics further show that older adults with dementia in Taiwan frequently have multiple chronic diseases and make extensive use of medical resources (Huang et al., 2024). In terms of caregiving, Behavioral and Psychological Symptoms of Dementia (BPSD, also referred to as neuropsychiatric symptoms)—particularly **depression**, **agitation/aggression**, **apathy**, and **nocturnal sleep disturbances**—often consume more resources than cognitive symptoms and impose greater stress on caregivers (Feast et al., 2016).

The diagnosis and assessment of BPSD rely mainly on clinical interviews or questionnaires (Stella, 2013). Because patients with BPSD typically cannot accurately recall or describe their symptoms, these questionnaires are primarily completed by caregivers (Cummings, 2020; Mao et al., 2015). In Taiwan, where 85% of caregivers are non-professional family caregivers, many are unable to provide precise descriptions of the patient’s BPSD (Cotton & Verghese, 2024). This inaccuracy affects clinical evaluation and subsequent treatment planning. For example, when clinicians cannot determine whether BPSD has improved, they may continue long-term prescriptions of sedative-hypnotics that increase fall risk, or resort to off-label antipsychotics that may elevate cardiovascular and mortality risks in dementia patients (Huang et al., 2024).

The dependence on high-risk, non-indicated medications also reflects the limited understanding of the mechanisms underlying BPSD. Some studies suggest that amyloid and tau pathology involved in Alzheimer’s disease may contribute to BPSD (Ehrenberg et al., 2018). However, a growing body of research indicates that BPSD may not arise from a single mechanism; physical illnesses, sensory impairments—especially hearing and vision—and environmental factors such as lighting conditions may also trigger or exacerbate symptoms (Corbett et al., 2013; Guu et al., 2022). Therefore, identifying precipitating and aggravating factors should take precedence over treatment (Kales et al., 2015).

If wearable devices can be accepted and consistently worn by older adults with dementia, they could bring transformative changes to both assessment and treatment, while deepening our understanding of dementia progression and BPSD mechanisms. In terms of assessment, my recent research in the United Kingdom found that even individuals with severe dementia and

prominent agitation/aggression can successfully wear the research-grade Geneactiv actigraph continuously for more than four weeks, and that BPSD severity does not affect device acceptance or adherence (Guu et al., 2024). Research-grade devices like Geneactiv not only enable validated, open-algorithm sleep assessment (van Hees et al., 2015) but also allow exploration of long-term dynamic behavioral patterns—such as the Sleep Regularity Index, which is strongly inversely related to cardiovascular disease and shows a U-shaped relationship with dementia risk—and may help assess agitation, sleep disturbances, and even the influence of light exposure on BPSD (Guu, 2024).

On the treatment side, previous studies have identified insufficient environmental light exposure as a potential cause of BPSD in people with dementia, especially among those in long-term care facilities with limited outdoor activity (Guu et al., 2022). Although certain wavelengths of environmental lighting and light-based therapies may help alleviate depression and nocturnal sleep disturbances, evidence has been mixed (Hjetland et al., 2021; Hjetland et al., 2020; Kolberg et al., 2021). Potential explanations include the distance between environmental lighting and the eyes, as well as age-related declines in visual light reception, which may reduce therapeutic effectiveness (Guu et al., 2022).

"Re-Timer" (http://re-timer.com/) is a glasses-type wearable device that emits 500-nm blue-green light. Preliminary studies suggest that it may improve depression, circadian rhythm regulation, and sleep symptoms in adults (Corbett, 2013; Lovato & Lack, 2016; Zalta et al., 2019). A pilot study also found that older adults with mild depressive symptoms and sleep disturbances could wear the device daily for 30 minutes over two weeks, and that their sleep rhythms may respond to the intervention (Leggett et al., 2018). If older adults with dementia could wear such devices consistently for longer periods, this non-pharmacological approach might alleviate their BPSD, reduce exposure to high-risk medications, and—if caregivers also use the device—potentially relieve caregiver stress as well.

Research Aims:

1. Primary:
To determine the **acceptability** and **feasibility of long-term daily use** of two different wearable devices—**Geneactiv** and **Re-Timer**—among dementia patients with significant BPSD and their caregivers.

2. Secondary:
To obtain preliminary information regarding the **effects of these devices on BPSD and sleep symptoms**, in order to support the estimation of sample size and study duration for subsequent trials. Additionally, to gain an initial understanding of the relationship between **light exposure, circadian rhythms**, and the **occurrence and fluctuations of BPSD**.

Importance:

1. To determine the acceptability and applicability of wearable devices among dementia patients with significant BPSD living in rural areas of Taiwan, as well as their caregivers, and to obtain representative, Taiwan-specific data (Guu et al., 2023). These findings will support the development of new care models—using wearable devices for the assessment and treatment of BPSD—as Taiwan transitions into a super-aged society.

2. To provide more objective evidence for differentiating between daytime and nighttime BPSD (Cummings et al., 2024), thereby helping clinical care providers more comprehensively consider the impact of environmental factors and daily lifestyle on human behavior, ultimately enabling the development of more personalized treatment plans.

References:

Buysse, D. J., Reynolds, C. F., 3rd, Monk, T. H., Berman, S. R., & Kupfer, D. J. (1989). The Pittsburgh Sleep Quality Index: a new instrument for psychiatric practice and research. *Psychiatry Res*, *28*(2), 193-213. https://doi.org/10.1016/0165-1781(89)90047-4

Carrillo, M. C., Dean, R. A., Nicolas, F., Miller, D. S., Berman, R., Khachaturian, Z., Bain, L. J., Schindler, R., & Knopman, D. (2013). Revisiting the framework of the National Institute on Aging-Alzheimer's Association diagnostic criteria. *Alzheimer's & Dementia*, *9*(5), 594-601. https://doi.org/https://doi.org/10.1016/j.jalz.2013.05.1762

Chou, K. R., Jiann-Chyun, L., & Chu, H. (2002). The reliability and validity of the Chinese version of the caregiver burden inventory. *Nurs Res*, *51*(5), 324-331. https://doi.org/10.1097/00006199-200209000-00009

Corbett, A., Nunez, K., & Thomas, A. (2013). Coping with dementia in care homes. *Maturitas*, *76*(1), 3-4. https://doi.org/10.1016/j.maturitas.2013.06.002

Corbett, M. A. (2013). A potential aid to circadian adaptation: re-timer. *Aviat Space Environ Med*, *84*(10), 1113-1114. https://doi.org/10.3357/asem.3827.2013

Cotton, K., & Verghese, J. (2024). Dementia in Taiwan. *Archives of Gerontology and Geriatrics*, *121*, 105415. https://doi.org/https://doi.org/10.1016/j.archger.2024.105415

Cummings, J. (2020). The Neuropsychiatric Inventory: Development and Applications. *J Geriatr Psychiatry Neurol*, *33*(2), 73-84. https://doi.org/10.1177/0891988719882102

Cummings, J., Sano, M., Auer, S., Bergh, S., Fischer, C. E., Gerritsen, D., Grossberg, G., Ismail, Z., Lanctôt, K., Lapid, M. I., Mintzer, J., Palm, R., Rosenberg, P. B., Splaine, M., Zhong, K., & Zhu, C. W. (2024). Reduction and prevention of agitation in persons with neurocognitive disorders: an international psychogeriatric association consensus algorithm. *Int Psychogeriatr*, *36*(4), 251-262. https://doi.org/10.1017/s104161022200103x

Ehrenberg, A. J., Suemoto, C. K., Franca Resende, E. P., Petersen, C., Leite, R. E. P.,

Rodriguez, R. D., Ferretti-Rebustini, R. E. L., You, M., Oh, J., Nitrini, R., Pasqualucci, C. A., Jacob-Filho, W., Kramer, J. H., Gatchel, J. R., & Grinberg, L. T. (2018). Neuropathologic Correlates of Psychiatric Symptoms in Alzheimer's Disease. *J Alzheimers Dis*, *66*(1), 115-126. https://doi.org/10.3233/JAD-180688

Farina, N., Sherlock, G., Thomas, S., Lowry, R. G., & Banerjee, S. (2019). Acceptability and feasibility of wearing activity monitors in community-dwelling older adults with dementia. *Int J Geriatr Psychiatry*, *34*(4), 617-624. https://doi.org/10.1002/gps.5064

Feast, A., Moniz-Cook, E., Stoner, C., Charlesworth, G., & Orrell, M. (2016). A systematic review of the relationship between behavioral and psychological symptoms (BPSD) and caregiver well-being. *Int Psychogeriatr*, *28*(11), 1761-1774. https://doi.org/10.1017/s1041610216000922

Guu, T.-W., Brem, A.-K., Albertyn, C. P., Kandangwa, P., Aarsland, D., & ffytche, D. (2024). Wrist-worn actigraphy in agitated late-stage dementia patients: A feasibility study on digital inclusion. *Alzheimer's & Dementia*, *20*(5), 3211-3218. https://doi.org/https://doi.org/10.1002/alz.13772

Guu, T. W. (2024). *STAND-S study - explore sleep, circadian rhythm, light and neuropsychiatry symptoms in dementia with wearable devises* King's College London]. Institute of Psychiatry, Psychology and Neuroscience. https://kclpure.kcl.ac.uk/portal/en/studentTheses/stand-s-study-explore-sleep-circadian-rhythm-light-and-neuropsych

Guu, T. W., Aarsland, D., & Ffytche, D. (2022). Light, sleep-wake rhythm, and behavioural and psychological symptoms of dementia in care home patients: Revisiting the sundowning syndrome. *Int J Geriatr Psychiatry*, *37*(5). https://doi.org/10.1002/gps.5712

Guu, T. W., Muurling, M., Khan, Z., Kalafatis, C., Aarsland, D., Ffytche, D., & Brem, A. K. (2023). Wearable devices: underrepresentation in the ageing society. *Lancet Digit Health*, *5*(6), e336-e337. https://doi.org/10.1016/s2589-7500(23)00069-9

Hjetland, G. J., Kolberg, E., Pallesen, S., Thun, E., Nordhus, I. H., Bjorvatn, B., & Flo-Groeneboom, E. (2021). Ambient bright light treatment improved proxy-rated sleep but not sleep measured by actigraphy in nursing home patients with dementia: a placebo-controlled randomised trial. *BMC Geriatr*, *21*(1), 312. https://doi.org/10.1186/s12877-021-02236-4

Hjetland, G. J., Pallesen, S., Thun, E., Kolberg, E., Nordhus, I. H., & Flo, E. (2020). Light interventions and sleep, circadian, behavioral, and psychological disturbances in dementia: A systematic review of methods and outcomes. *Sleep Med Rev*, *52*, 101310. https://doi.org/10.1016/j.smrv.2020.101310

Huang, S.-T., Loh, C.-H., Lin, C.-H., Hsiao, F.-Y., & Chen, L.-K. (2024). Trends in dementia incidence and mortality, and dynamic changes in comorbidity and healthcare utilization from 2004 to 2017: A Taiwan national cohort study. *Archives of Gerontology and Geriatrics*, *121*, 105330. https://doi.org/https://doi.org/10.1016/j.archger.2024.105330

Jack, C. R., Jr., Albert, M. S., Knopman, D. S., McKhann, G. M., Sperling, R. A., Carrillo, M. C., Thies, B., & Phelps, C. H. (2011). Introduction to the recommendations from the National Institute on Aging-Alzheimer's Association workgroups on diagnostic

guidelines for Alzheimer's disease. *Alzheimers Dement*, *7*(3), 257-262. https://doi.org/10.1016/j.jalz.2011.03.004

Kales, H. C., Gitlin, L. N., & Lyketsos, C. G. (2015). Assessment and management of behavioral and psychological symptoms of dementia. *BMJ*, *350*, h369. https://doi.org/10.1136/bmj.h369

Kolberg, E., Hjetland, G. J., Thun, E., Pallesen, S., Nordhus, I. H., Husebo, B. S., & Flo-Groeneboom, E. (2021). The effects of bright light treatment on affective symptoms in people with dementia: a 24-week cluster randomized controlled trial. *BMC Psychiatry*, *21*(1), 377. https://doi.org/10.1186/s12888-021-03376-y

Leggett, A. N., Conroy, D. A., Blow, F. C., & Kales, H. C. (2018). Bright Light as a Preventive Intervention for Depression in Late-Life: A Pilot Study on Feasibility, Acceptability, and Symptom Improvement. *Am J Geriatr Psychiatry*, *26*(5), 598-602. https://doi.org/10.1016/j.jagp.2017.11.007

Lovato, N., & Lack, L. (2016). Circadian phase delay using the newly developed re-timer portable light device. *Sleep and Biological Rhythms*, *14*(2), 157-164. https://doi.org/10.1007/s41105-015-0034-6

Lunsford-Avery, J. R., Engelhard, M. M., Navar, A. M., & Kollins, S. H. (2018). Validation of the Sleep Regularity Index in Older Adults and Associations with Cardiometabolic Risk. *Sci Rep*, *8*(1), 14158. https://doi.org/10.1038/s41598-018-32402-5

Lyketsos, C. G., Lopez, O., Jones, B., Fitzpatrick, A. L., Breitner, J., & DeKosky, S. (2002). Prevalence of neuropsychiatric symptoms in dementia and mild cognitive impairment: results from the cardiovascular health study. *JAMA*, *288*(12), 1475-1483. https://doi.org/10.1001/jama.288.12.1475

Mao, H. F., Chen, W. Y., Yao, G., Huang, S. L., Lin, C. C., & Huang, W. N. (2010). Cross-cultural adaptation and validation of the Quebec User Evaluation of Satisfaction with Assistive Technology (QUEST 2.0): the development of the Taiwanese version. *Clin Rehabil*, *24*(5), 412-421. https://doi.org/10.1177/0269215509347438

Mao, H. F., Kuo, C. A., Huang, W. N., Cummings, J. L., & Hwang, T. J. (2015). Values of the Minimal Clinically Important Difference for the Neuropsychiatric Inventory Questionnaire in Individuals with Dementia. *J Am Geriatr Soc*, *63*(7), 1448-1452. https://doi.org/10.1111/jgs.13473

Stella, F. (2013). Assessment of neuropsychiatric symptoms in dementia: toward improving accuracy. *Dement Neuropsychol*, *7*(3), 244-251. https://doi.org/10.1590/S1980-57642013DN70300003

van Hees, V. T., Sabia, S., Anderson, K. N., Denton, S. J., Oliver, J., Catt, M., Abell, J. G., Kivimaki, M., Trenell, M. I., & Singh-Manoux, A. (2015). A Novel, Open Access Method to Assess Sleep Duration Using a Wrist-Worn Accelerometer. *PLoS One*, *10*(11), e0142533. https://doi.org/10.1371/journal.pone.0142533

Zalta, A. K., Bravo, K., Valdespino-Hayden, Z., Pollack, M. H., & Burgess, H. J. (2019). A placebo-controlled pilot study of a wearable morning bright light treatment for probable PTSD. *Depress Anxiety*, *36*(7), 617-624. https://doi.org/10.1002/da.22897

D. Research Methods and Procedures

1. Please describe in detail the research methods adopted in this project and the rationale for choosing them.
2. Describe the potential challenges anticipated during the project and the strategies for addressing them.
3. Explain the use and coordination of major instruments or equipment.
4. For projects lasting more than one year, please outline the plan year by year.
5. If this project is an integrated (multi-component) project, please address each of the above points and explain its relevance to the other sub-projects.

Research Methods Overview

This study will recruit dementia patients who exhibit at least one of four prominent Behavioral and Psychological Symptoms of Dementia (BPSD)—sleep disturbance, agitation, apathy, or depression—from the Psychiatry and Neurology outpatient clinics of Beigang Hospital, as well as from the affiliated Yuanchang Dementia Day-Care Center and five community dementia care hubs. Their primary caregivers will also be invited to participate. Members of the care team (including physicians, dementia case managers, or nurses) will first ask patients and caregivers whether they are willing to join the study. Those who express willingness will subsequently receive a detailed explanation from the principal investigator and undergo the corresponding intervention procedures.

The study design is a single-arm dyadic pilot study. Both patients and caregivers will wear the Re-Timer optical light-therapy glasses for four weeks, at least 30 minutes per day, and will wear the Geneactiv actigraph for a total of eight weeks—two weeks before, four weeks during, and two weeks after the Re-Timer intervention.

Study Participants

1. Planned Enrollment: A total of 10 patients and their 10 primary caregivers.

2. Inclusion Criteria

(1) Patients

a. Clinical diagnosis of probable Alzheimer's disease (Probable AD), or mild cognitive impairment due to AD with a CDR score of 0.5 (Carrillo et al., 2013; Jack et al., 2011).
b. Presence of at least one "significant" BPSD symptom among depression, agitation, apathy, or nocturnal sleep disturbance.

— "Significant" is defined as:

• Neuropsychiatric Inventory (NPI) item score ≥ 4 (severity $\times$ frequency ≥ 4) (Lyketsos et al., 2002), or

• NPI-Q severity score ≥ 2;

• If sleep disturbance is the primary symptom, Pittsburgh Sleep Quality Index (PSQI) total score ≥ 5 (Buysse et al., 1989).

c. Stable residence in the current setting for ≥ 2 weeks
(e.g., community participants must have lived at the same residence ≥ 2 weeks; day-care participants must have attended ≥ 2 weeks).
d. If currently receiving medication or non-pharmacological treatment for BPSD, the regimen must have been stable for ≥ 2 weeks prior to enrollment.
e. Meets none of the exclusion criteria and provides informed consent.
If unable to consent due to cognitive decline, consent must be provided by a legally authorized representative.

(2) Primary Caregivers

a. Adult primary caregiver of the enrolled patient.
b. If receiving treatment for caregiving stress, the regimen must have been stable for ≥ 2 weeks prior to enrollment.
c. Meets none of the exclusion criteria and signs informed consent.

3. Exclusion Criteria

(1) Individuals who may be harmed by the intervention, including those with retinal disease, those taking photosensitizing medications, those who have undergone ocular surgery within 4 weeks, or those with other medical/psychological conditions unsuitable for blue-green light exposure (e.g., epilepsy).
(2) Individuals clinically assessed as medically or psychologically unstable, such as those experiencing acute delirium or acute respiratory infections (including COVID).

Research Equipment

1. Geneactiv Actigraph

The Geneactiv device resembles a wristwatch and includes a light sensor that detects ambient light intensity. It is one of the few research-grade actigraphs validated in both adult and older adult populations. Although no studies have evaluated its use among Taiwanese dementia populations, recent research—including studies conducted by the PI in the UK—has shown that both community-dwelling individuals with mild dementia and institutionalized individuals with severe dementia can accept and consistently wear the device (Farina et al., 2019; Guu et al., 2024).

Research personnel will assist with fitting the device at study initiation. Based on the PI's previous findings that design and aesthetics may influence device adherence among dementia patients (Guu et al., 2024), manufacturer-approved replacement straps will be offered. Participants will be instructed to remove the device if discomfort occurs and to avoid covering the light sensor with clothing.

2. Re-Timer Circadian Regulator

Re-Timer is a glasses-type light-therapy device that emits 500-nm blue-green light, free of ultraviolet radiation, and is not regulated as a medical device under Taiwan FDA classification. Its light intensity (illuminance 506 Lux Im/m²; irradiance 230 μW/cm²) has been validated in early studies involving healthy adults, adults with psychiatric conditions, and older adults (Corbett, 2013; Leggett et al., 2018; Lovato & Lack, 2016; Zalta et al., 2019).

Research personnel will verify correct wearing position to ensure light reaches the pupil area. Participants may remove the device at any time if discomfort occurs.

Study Procedures and Interventions

1. All participants will begin wearing the Geneactiv actigraph on Day 1 and continue until the end of Week 8 (Day 56), for a total of eight weeks.
2. Outpatient dementia patients and all caregivers who agree to use Re-Timer will begin wearing the device on Day 15 through Day 42 (four weeks), for 30–60 minutes per day, recording wear time.
   Day-care center and dementia-hub participants will follow the same schedule (Day 15–42), with the option to:
   - wear Re-Timer only during center activities with staff assistance, or
   - bring the device home for daily use.
3. If any discomfort occurs during device use, participants may stop wearing the device immediately. Research personnel will assist in documenting symptoms and arranging medical referral if needed. Participants may withdraw from the study when necessary.

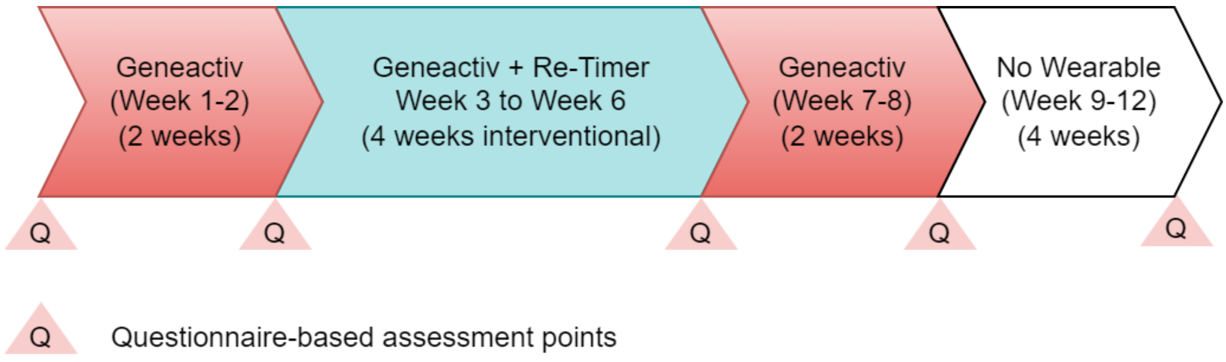

Figure 1. Study Flowchart (Narrative Description)

The study spans **12 weeks**. Participants will wear the **Geneactiv actigraph for up to 8 weeks** and the **Re-Timer device for up to 4 weeks**, and will complete **five rounds of questionnaire assessments** on Day 1, Day 15, Day 42, Day 56, and Day 84 (assessment items described in "Assessment Items and Methods").

Assessment Items and Methods

1. Collection of Basic Information

(1) Dementia Patients

Data collected will include:

- date of birth, sex, marital status, educational level
- date of dementia (or MCI) diagnosis
- current cognitive status (CDR, CASI, or MMSE within the past 6 months; if noticeable recent decline is suspected, reassessment will be performed and the most recent result recorded)
- onset time of BPSD
- current dementia-related medications
- smoking and alcohol use
- medical and psychiatric history and current treatments

(2) Caregivers

Data collected will include:

- date of birth, sex, marital status, educational level
- smoking and alcohol use
- medical and psychiatric history and current treatments

If the caregiver is an older adult with possible cognitive decline, cognitive assessment (CDR, CASI, or MMSE within 6 months; repeated if recent decline is suspected) will also be collected.

2. Questionnaire Assessments

(1) Pittsburgh Sleep Quality Index (PSQI) (Buysse et al., 1989)

Assesses seven domains of sleep. Each item ranges from 0 (no symptom) to 3.
Administered **five times**: Day 1, Day 15, Day 42, Day 56, Day 84.
Both caregiver and patient complete the PSQI.
If the patient is unable to self-report, the caregiver may complete it on their behalf.

(2) Chinese Version of the Neuropsychiatric Inventory Questionnaire (NPI-Q) (Mao et al., 2015)

Assesses 12 behavioral symptoms:

- presence,
- severity (1–3),
- caregiver distress (0–5).

Administered **five times** on the same schedule as above.

Each patient is consistently rated by **the same primary caregiver**.

(3) Chinese Version of the Caregiver Burden Inventory (CBI) (Chou et al., 2002)

24 items across five domains (0–4 per item). Higher scores indicate greater burden.
Administered **three times**: Day 1, Day 42, Day 84.
Completed by the same primary caregiver throughout the study.

(4) Taiwanese Version of the Quebec User Evaluation of Satisfaction with Assistive Technology (T-QUEST) (Mao et al., 2010)

13 items scored from 1 (“very dissatisfied”) to 5 (“very satisfied”).
Administered to both caregivers and patients.

- **Day 42:** Satisfaction with **Re-Timer**
- **Day 56:** Satisfaction with **Geneactiv**

If a patient cannot complete the questionnaire, only the caregiver who used the device will respond.

3. Semi-Structured Interviews

To deepen understanding of device acceptability, usability, comfort, and perceived challenges, qualitative interviews will extend the content of the T-QUEST.

- Each interview lasts **30–60 minutes**.
- Conducted in an environment comfortable for the participant and caregiver.
- Audio recordings will be transcribed verbatim.
- Analysis will follow **thematic analysis**, aiming to understand perceptions of using wearable devices for psychiatric assessment and treatment after participating in the trial.

Data Analysis and Presentation

1. General Approach

All statistical analyses will be performed using **R Studio and R**.
Descriptive statistics will summarize demographic characteristics using means, percentages, and standard deviations as appropriate.

2. Primary Outcome Analysis: Acceptability and Feasibility

A mixed quantitative–qualitative approach will be used.

(1) Quantitative Analysis

- Wear time for Geneactiv will be calculated using the validated open-source package **GGIR** (van Hees et al., 2015).

- Wear adherence = actual wear time ÷ total possible wear time.
- Re-Timer wear time will be recorded daily by the user (or by caregivers for patients).
  - Adherence = total recorded wear time ÷ total possible wear time.
- T-QUEST total and subscale scores will quantify satisfaction and acceptability.
- Differences between dementia patients and caregivers will be analyzed using **independent t-tests**.

(2) Qualitative Analysis

- All transcripts will be read thoroughly for initial coding.
- Codes will be refined into themes, identifying similarities and differences between patients and caregivers.
- **Triangulation** will be used:
  - independent coding by multiple researchers,
  - comparison and consensus to ensure reliability.
- Background factors (age, culture, disease stage, etc.) will be considered in interpreting perspectives.
- Findings will explore potential opportunities and barriers in using wearable devices for psychiatric assessment and treatment.

3. Secondary Outcome Analysis: Preliminary Treatment Effects

The study explores the preliminary effects of Re-Timer on:

- BPSD in dementia patients,
- sleep symptoms in both patients and caregivers.

Key variables extracted from wearable sensors (via GGIR) will include:

- activity levels
- sleep parameters (sleep onset, wake after sleep onset, wake time, total sleep time)
- **Sleep Regularity Index** (Lunsford-Avery et al., 2018)
- light-exposure metrics

These will be paired with NPI-Q and PSQI scores in a longitudinal time-series framework.

Analytical Models

- **Mixed-effects models** will analyze repeated measures and individual variability.
- Pre-post comparisons will evaluate whether wearable-based interventions improve

BPSD or sleep symptoms.

- Covariates (age, dementia severity, comorbidities) will be entered to control for confounding.
- Potential **dose–response** effects of light exposure and circadian regulation will be evaluated.

Sample Size Planning

- Preliminary findings will inform future power calculations.
- **Cohen's d** will be used to estimate effect sizes.
- Sample size will be calculated based on expected effect size, significance level, and desired statistical power.

Public–Patient Involvement and Engagement (PPIE)

To ensure that the study design and results reflect the needs and perspectives of patients and caregivers, the following PPIE activities will be included:

1. Advisory Group and Consultation Meetings

An advisory group will be formed, consisting of dementia patients, caregivers, relevant NGO representatives, and healthcare professionals.
Meetings will:

- collect early feedback on wearable device use
- explore perceived acceptability and potential challenges
- discuss views on light exposure and circadian interventions
- integrate participant input into optimizing future study design

2. Result Interpretation and Knowledge Dissemination

After study completion:

- Patients and caregivers will be invited to result-interpretation meetings to review and discuss findings.
- These meetings will improve transparency and incorporate their lived experiences into interpretation.
- Public education sessions will be held to share results in accessible language, promote knowledge translation, and encourage future participation.

E. Expected Work Items and Deliverables

1. Expected Work Items to Be Completed During the Project Period. Please list the work items expected to be completed within the project execution period.
2. Expected Contributions to Academic Research, National Development, and Other Applications. Please describe how the project's outcomes will contribute to academic advancement, national development, and practical or clinical applications.
3. Expected Training and Skills Gained by Participating Personnel. Please explain the types of training, professional development, and research skills that project staff members are expected to acquire through participation in this project.
4. For Projects Exceeding One Year, Please Describe Plans Separately by Year. For multi-year projects, present the planned goals, work content, and milestones for each year.
5. For Sub-projects Under an Integrated Project, Please Explain Each of the Above Points in Relation to Other Sub-projects. If this proposal is submitted as part of an integrated project, explain how each of the above aspects relates to and coordinates with the other sub-projects.

Expected Benefits Based on the Research Aims

1. **Improving the Application of Wearable Devices in Patients with BPSD**

   By evaluating the acceptability and feasibility of the Geneactiv and Re-Timer devices, this study will help optimize the design and practical use of these technologies. The findings will contribute to increasing the willingness of people with dementia and their caregivers to use wearable devices consistently in daily life.

2. **Collection of Preliminary Efficacy Data**

   The study will generate preliminary evidence on the effects of Re-Timer on BPSD and sleep disturbances. These data will help determine whether the device can improve the well-being of patients and caregivers, while also providing direction and foundational information for future larger-scale studies.

3. **Understanding the Impact of Light Exposure and Circadian Rhythms on BPSD**

   By analyzing long-term activity patterns and light-exposure data captured by Geneactiv, this study will explore the relationship between light, circadian rhythms, and changes in BPSD. These findings may yield clinically meaningful insights into new non-pharmacological intervention strategies and support improvements in both the assessment and treatment of BPSD.

F. Planned Progress Gantt Chart

1. This table serves as the basis for evaluating progress and project-management milestones.
2. Work Items:
   Please define work items according to the nature and requirements of the project.
   Indicate the planned schedule using thick horizontal bars to show the start and end months.
   Each month is divided into three ten-day periods.
   For projects constrained by agricultural or other seasonal factors, please specify the actual applicable months under the "Month" column to facilitate review.
3. Estimated Cumulative Progress (%):
   For progress-tracking and evaluation purposes, please estimate cumulative progress using one of the following factors—selected according to the characteristics of the work:
   (1) number of working days
   (2) distribution of budget
   (3) relative weight or importance of the task
   (4) specific numerical targets expected to be achieved
4. For projects extending beyond one year, please prepare a separate Gantt chart for each year.
5. If this project is a sub-project within an integrated program, please explain each of the above items and describe how they relate to the other sub-projects.

Task \ Month	1-2	3-4	5-6	7-8	9-10	11-12	13-14	15-16	17-18	19-20	21-22	23-24	Note
Deployment and testing of research equipment	······	······											
Ethical and administrative approvals	······	······	······										
Study announcement and participant recruitment				······	······	······	······						
Participant data collection				······	······	······	······	······					
Final semi-structured qualitative interviews									······	······			
Overall data cleaning and analyses									······	······			
Results and Manuscript preparation									······	······	······	······	
Public and patient involvements and engagements											······	······	
Estimated cumulative progress (%)	5	10	15	20	30	40	50	60	70	80	90	100	
